# Metabolic analysis of kiwifruit (*Actinidia deliciosa*) berries from extreme genotypes reveals hallmarks for fruit starch metabolism

**DOI:** 10.1093/jxb/ert293

**Published:** 2013-09-21

**Authors:** Simona Nardozza, Helen L. Boldingh, Sonia Osorio, Melanie Höhne, Mark Wohlers, Andrew P. Gleave, Elspeth A. MacRae, Annette C. Richardson, Ross G. Atkinson, Ronan Sulpice, Alisdair R. Fernie, Michael J. Clearwater

**Affiliations:** ^1^The New Zealand Institute for Plant & Food Research Limited (PFR), Mt Albert Research Centre, Private Bag 92169, Auckland, New Zealand; ^2^PFR, Ruakura Research Centre, Private Bag 3230, Hamilton, New Zealand; ^3^Max Planck Institute of Molecular Plant Physiology, Wissenschaftspark Golm, Am Mühlenberg 1, D-14476 Potsdam-Golm, Germany; ^4^PFR, Kerikeri Research Centre, Private Bag 23, Kerikeri, New Zealand; ^5^PFR, Te Puke Research Centre, 412 No 1 Road, RD 2, Te Puke, New Zealand

**Keywords:** *Actinidia deliciosa*, AGPase, berry development, enzyme profiling, fruit quality, glucose, metabolite profiling, neutral invertase, planteose, primary metabolism, starch, sucrose phosphate synthase, transcript profiling.

## Abstract

Tomato, melon, grape, peach, and strawberry primarily accumulate soluble sugars during fruit development. In contrast, kiwifruit (*Actinidia* Lindl. spp.) and banana store a large amount of starch that is released as soluble sugars only after the fruit has reached maturity. By integrating metabolites measured by gas chromatography–mass spectrometry, enzyme activities measured by a robot-based platform, and transcript data sets during fruit development of *Actinidia deliciosa* genotypes contrasting in starch concentration and size, this study identified the metabolic changes occurring during kiwifruit development, including the metabolic hallmarks of starch accumulation and turnover. At cell division, a rise in glucose (Glc) concentration was associated with neutral invertase (NI) activity, and the decline of both Glc and NI activity defined the transition to the cell expansion and starch accumulation phase. The high transcript levels of β-amylase 9 (*BAM9*) during cell division, prior to net starch accumulation, and the correlation between sucrose phosphate synthase (SPS) activity and sucrose suggest the occurrence of sucrose cycling and starch turnover. ADP-Glc pyrophosphorylase (AGPase) is identified as a key enzyme for starch accumulation in kiwifruit berries, as high-starch genotypes had 2- to 5-fold higher AGPase activity, which was maintained over a longer period of time and was also associated with enhanced and extended transcription of the AGPase large subunit 4 (*APL4*). The data also revealed that SPS and galactinol might affect kiwifruit starch accumulation, and suggest that phloem unloading into kiwifruit is symplastic. These results are relevant to the genetic improvement of quality traits such as sweetness and sugar/acid balance in a range of fruit species.

## Introduction

Kiwifruit (*Actinidia* Lindl. spp.) is one of the few new fruit crops successfully adopted by international markets in recent years. It has become a high value export crop for a number of countries including New Zealand, Italy, and Chile. The success of kiwifruit has been largely based on a single cultivar, *Actinidia deliciosa* ‘Hayward’, which has a distinctive appearance and flavour, compared with other fruit types, as well as health benefits from high concentrations of vitamin C and fibre (Ferguson and Ferguson, 2003). In the past decade, several new kiwifruit cultivars have been developed and released to complement sales of ‘Hayward’ fruit in world markets. However, to accelerate the development of further new cultivars, a greater understanding of the metabolic control of major quality traits in kiwifruit is required.

Sweetness is the single most important quality trait for kiwifruit as it influences overall fruit flavour (sugar/acid balance, perception of volatiles), consumer acceptability, and grower returns (Harker *et al.*, 2009; Nardozza *et al.*, 2011*a*). While sucrose (Suc) and planteose are the major sugars transported to fruit during development (Klages *et al.*, 2004), adequate storage of soluble sugars as starch/dry matter (DM) reserves is essential to produce an acceptably flavoured kiwifruit berry (Richardson *et al.*, 1997). Starch metabolism in kiwifruit is a complex, dynamic process, characterized by simultaneous starch accumulation and degradation (Wegrzyn and MacRae, 1995). Net starch accumulation does not occur until the fruit has completed cell division (Richardson *et al.*, 2004), while net starch degradation mainly occurs after fruit harvest and, by the time the fruit is eating ripe, almost all starch has been converted into soluble sugars (MacRae *et al.*, 1992).

Metabolic profiling has been applied to study developmental changes in several fruits, such as tomato (*Solanum lycopersicum* L.) (Carrari *et al.*, 2006; Steinhauser *et al.*, 2010), grape (*Vitis vinifera* L.) (Deluc *et al.*, 2007; Dai *et al.*, 2013), strawberry [*Fragaria×ananassa* (Weston) Duchesne ex Rozier (pro sp.)] (Fait *et al.*, 2008), melon (*Cucumis* L.) (Dai *et al.*, 2011), and peach [*Prunus persica* (L.) Batsch] (Lombardo *et al.*, 2011). All these fruits have been demonstrated to differ from one another not only from physiological and botanical points of view, but also at the metabolic level. What was clearly identified in these studies was the need to extend metabolite investigations to a wide range of fruit species, especially those where the metabolism differs significantly from the tomato model. Kiwifruit vines bear fleshy fruit that are botanically classified as berries and therefore share characteristics with the model berry, tomato. However, kiwifruit differs from tomato in several important ways. Kiwifruit accumulate large amounts of starch over a long period of time, store only limited amounts of soluble sugars, and the development of osmotic pressure is mainly driven by organic acid accumulation (Nardozza *et al.*, 2010). Kiwifruit is also different from grape, melon, and apple (*Malus* Mill.) which accumulate all or most carbon as soluble sugars (Deluc *et al.*, 2007; Dai *et al.*, 2011; Li *et al.*, 2012), and from peach and strawberry which do not produce berries (being a stone fruit and an aggregate accessory fruit, respectively), and also do not accumulate significant amounts of starch (Fait *et al.*, 2008; Lombardo *et al.*, 2011). Current knowledge of kiwifruit primary metabolism is limited to the commercial cultivars ‘Hayward’ and ‘Hort16A’ (Richardson *et al.*, 2011; Moscatello *et al.*, 2011).

This comprehensive analysis of the Suc to starch metabolic pathway during kiwifruit development combines data on primary metabolites, transcripts, and enzyme activities to identify changes between developmental stages and regulatory steps that affect carbohydrate accumulation. This work examines the hallmarks of starch accumulation in the kiwifruit berry by investigating why certain kiwifruit genotypes are able to accumulate twice the amount of starch during fruit development compared with others (Nardozza *et al.*, 2010). The majority of fruit metabolic studies published to date, for any crop, focus on one cultivar. The novel approach adopted in this work was to compare genotypes contrasting in fruit size and starch concentration. The results show that ADP-glucose pyrophosphorylase (AGPase) is a key enzyme involved in kiwifruit starch accumulation. Sucrose phosphate synthase (SPS) and galactinol (Gol) also appear to have roles in maximizing starch accumulation. Novel aspects of carbon metabolism in kiwifruit were also revealed, including that changes in glucose (Glc) levels and neutral invertase (NI) activity mark the transition to net starch accumulation, the presence of planteose and Gol in fruit, and the potential involvement of SPS/sucrose synthase (SUSY) and β-amylase 9/α-amylase 2 (*BAM9*/*AMY2*) in Suc cycling and starch turnover, respectively.

## Materials and methods

### Plant material, sampling, and physical measurements

*Actinidia deliciosa* (A. Chev.) C.F. Liang et A.R. Ferguson var. *deliciosa* genotypes characterized in this study were selected from a larger group of 24 genotypes (Nardozza *et al.*, 2010). In the 2007 harvest year (HY), fruit samples representative of all fruit tissue types were collected from 10 selected and replicated genotypes (Nardozza *et al.*, 2010) at four fruit developmental stages (Supplementary Tables S1, S2 available at *JXB* online), snap-frozen in liquid nitrogen, and stored at –80 °C for transcript analysis.

**Fig. 1.**
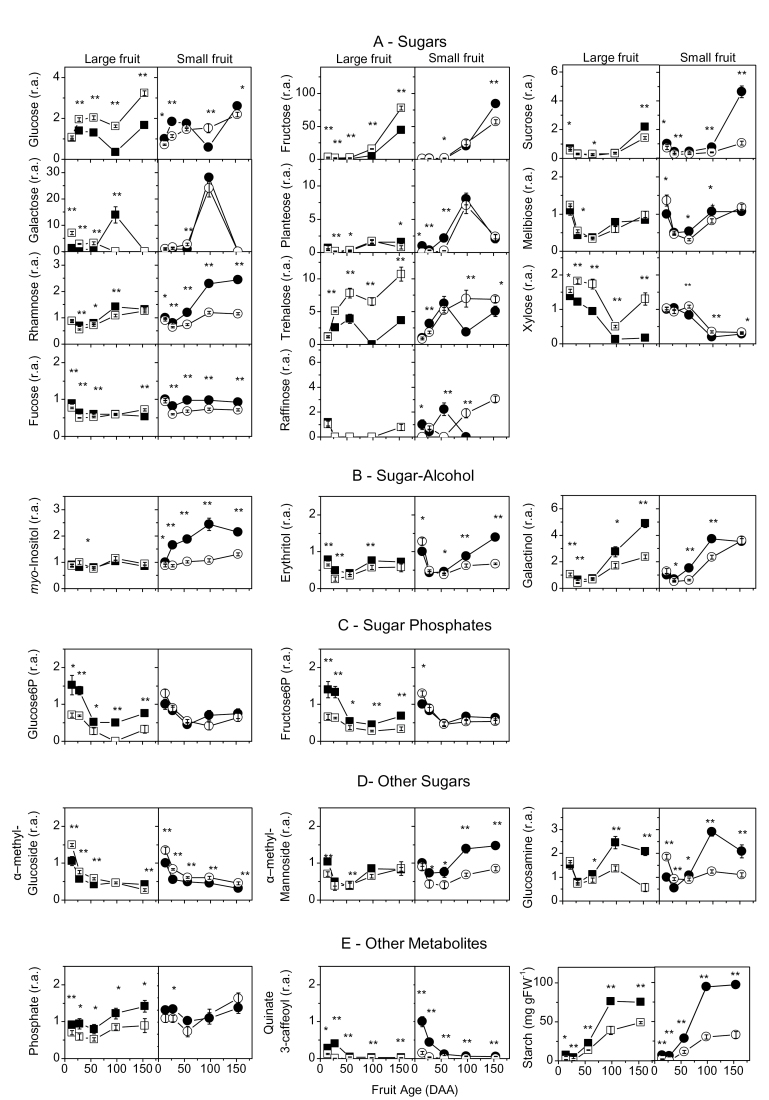

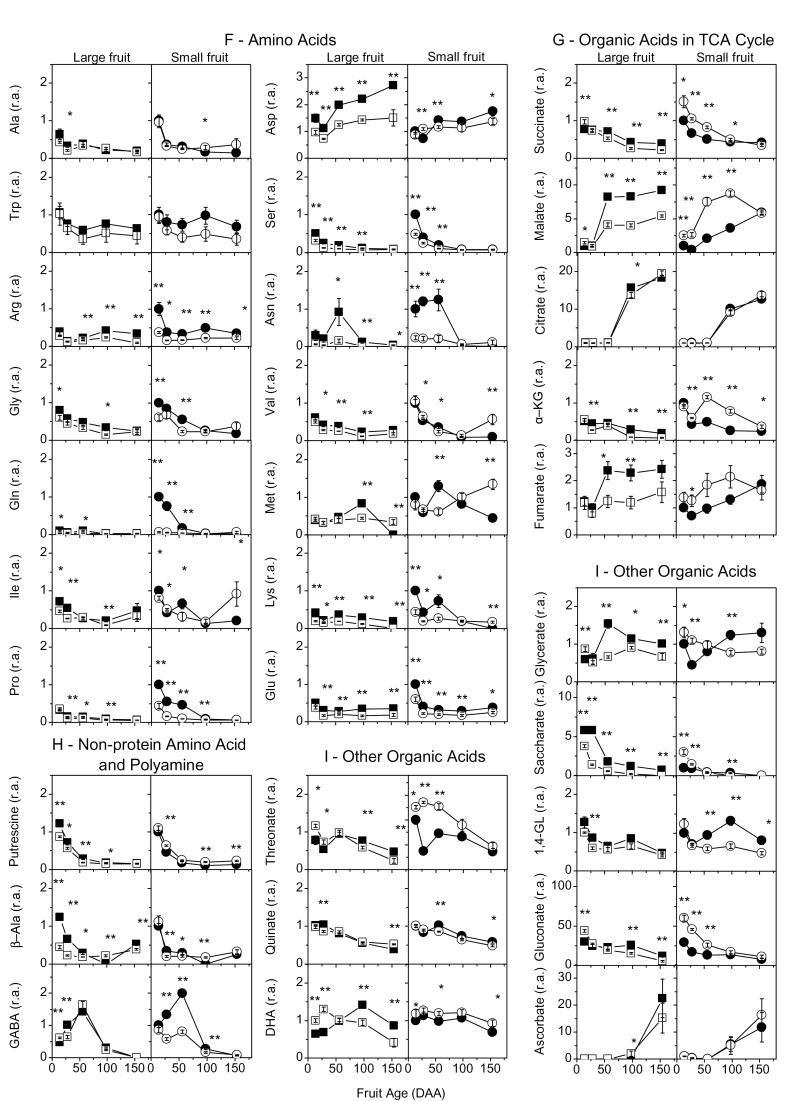
Changes in metabolite concentrations during fruit development of kiwifruit berries. Four genotypes were examined: G3, large fruit and high starch (filled squares); G25, large fruit and low starch (open squares); G30, small fruit and high starch (filled circles), and G17, small fruit and low starch (open circles). The 51 metabolites identified by GC-TOF-MS plus starch were subdivided into the following classes: A, sugars (Glc, Fru, sucrose, Gal, planteose, melibiose, Rha, Tre, Xyl, Fuc, raffinose); B, sugar-alcohols (*myo*-inositol, erythritol, galactinol); C, phosphate sugars (Glc6P, Fru6P); D, other sugars (α-methyl glucoside, α-methyl mannoside, glucosamine); E, other metabolites (phosphate, quinate 3-caffeoyl, starch); F, amino acids (alanine, tryptophan, arginine, glycine, glutamine, isoleucine, proline, aspartate, serine, asparagine, valine, methionine, lysine, glutamine); G, organic acids in the TCA cycle (succinate, malate, citrate, α-KG, fumarate); H, non-protein amino acids and polyamine (putrescine, β-alanine, GABA); I, other organic acids (threonate, quinate, DHA, gluconate, ascorbate, glycerate, saccharate, 1,4-GL). For each developmental stage, *t*-test significance levels are reported: *P < 0.05; **P < 0.01. Where the symbol is missing, the difference is not statistically significant. Fruit age is in days after anthesis (DAA); 1,4-GL, glucaric acid 1,4-lactone; DHA, dehydroascorbate; GABA, γ-aminobutyric acid; α-KG, α-ketoglutarate. Values are means of six biological replicates ±SEM. All data are expressed as relative amount (r.a.), with the exception of starch which is expressed as milligrams per gram fresh weight (mg g FW^–1^), and normalized to sample G30 at 14 DAA. Data refer to 2009 outer pericarp samples.

In the 2009 HY, a subset of four *A. deliciosa* genotypes was selected to give one individual each representing the extremes in starch concentration (high and low) and fruit size (large and small) (Fig. 1E; Supplementary Fig. S1B at *JXB* online). This experimental design minimizes the dilution effect of different rates of fresh weight (FW) growth that could hamper the identification of factors affecting starch accumulation. Fruit outer pericarp samples were collected in six biological replicates, at five different fruit developmental stages [14, 28, 56, 98, and 154 days after anthesis (DAA); Supplementary Tables S1, S3 at *JXB* online], snap-frozen in liquid nitrogen, and stored at –80 °C for metabolite, enzyme activity, and transcript analysis. FW, dry weight (DW), and DM were measured on sampled fruit as described in Nardozza *et al.* (2010).

The developmental stages were chosen to include key times when FW and starch curves were changing (Nardozza *et al.*, 2010). Supplementary Table S3 at *JXB* online shows their correspondence to the BBCH scale (Richardson *et al.*, 2011). Growth slowing (56 DAA and BBCH stage 75) represents the transition from cell division to cell expansion (Hopping, 1976).

### Profiling of primary metabolites

Polar metabolites were extracted following the method of Roessner *et al.* (2001) optimized for tomato fruit (Roessner-Tunali *et al.*, 2003). Samples were analysed by gas chromatography-time of flight-mass spectrometry (GC-TOF-MS) using a high-throughput platform and the protocol of Lisec *et al.* (2006). A 5 µl aliquot of extract was analysed to identify compounds present at high concentration [i.e. Glc, fructose (Fru), Suc, *myo-*inositol, citrate, quinate, and malate]. For metabolites at lower concentration, a second 50 µl aliquot was analysed. The retention time index standard mixture was a combination of fatty acid methyl esters (FAMEs). Mass spectra were cross-referenced with those of authentic standards in the Golm Metabolome Database (Kopka *et al.*, 2005; Schauer *et al.*, 2005). Metabolite quantification was based on the relative peak response area of each chromatogram (Fait *et al.*, 2008). The relative metabolite concentrations were determined on the basis of an internal standard (ribitol), normalized to sample G30 at 14 DAA and expressed as relative amounts (r.a.).

For soluble sugar (Glc, Fru, Suc, *myo*-inositol, planteose, and galactose) quantification, a third aliquot of extract was analysed by ion chromatography (IC). The methanol was blown off using a stream of nitrogen gas, and samples were redissolved in ultrapure water and analysed on a DIONEX ICS-3000 Reagent-Free™ IC (RFIC™) system with a CarboPac PA20 column (Trought *et al.*, 2011). Starch was analysed from the pellet obtained after the extraction of polar metabolites as described in Smith *et al.* (1992). Starch and sugar concentrations were expressed as milligrams per gram FW (mg g FW^ –1^).

### Carbohydrate and amino acid enzyme extractions and assays

Enzyme activities were measured by a semi-automated robot-based platform for multiple enzyme assays (Gibon *et al.*, 2004; Steinhauser *et al.*, 2010). With this sensitive method, a large number of enzyme activities can be measured from the same extract and no crude extract desalting is required.

Samples were ground in liquid nitrogen and aliquots (~40mg FW) of frozen powder were extracted after addition of 10mg of polyvinylpolypyrrolidone and vigorous vortexing in 500 µl of ice-cold extraction buffer (Supplementary Table S4 at *JXB* online). The crude extract was centrifuged for 10min at 3500rpm at 4 °C. Extract aliquots were further diluted with extraction buffer to obtain the optimal dilution for each enzyme (Supplementary Table S5 at *JXB* online). Enzyme activities were assayed immediately with established protocols (Gibon *et al.*, 2004; Steinhauser *et al.*, 2010) (Supplementary Table S6 at *JXB* online), giving higher priority to more unstable enzymes (i.e. SPS and SUSY).

To estimate the contribution of each measured enzyme in starch synthesis, the correlation coefficients of individual enzymes were calculated. Starch and enzyme values for all genotypes during net starch accumulation (56–154 DAA) were normalized on a zero to one scale. Then a regression analysis slope for each enzyme was calculated.

### Transcript analysis

RNA was isolated from 2g of fruit tissues as described in Chang *et al.* (1993) and DNase treated (DNA-free™; Ambion-Invitrogen). cDNA was synthesized using SuperScript™ III Reverse Transcriptase (Invitrogen). Gene-specific primers (Supplementary Table S7 at *JXB* online) were designed using Primer3 software (Rozen and Skaletsky, 2000) to a stringent set of criteria (Bulley *et al.*, 2009). Relative transcripts were quantified on a LightCycler 480 (Roche) real-time PCR detection system using a LightCycler 480 SYBR Green master mix (Roche) (Nieuwenhuizen *et al.*, 2009). Elongation factor 1α (*EF1α*) and protein phosphatase 2A (*PP2A*) were the housekeeping genes. Target gene transcripts were normalized to *PP2A* (Bulley *et al.*, 2009) using the 2^–ΔCt^ method (Ren *et al.*, 2007).

### Statistical analysis

Statistical analysis of the metabolite, IC sugar, starch, and the enzyme activity data was performed using Microsoft^®^ Excel two-samples *t*-tests assuming unequal variances. All the correlation analyses were performed in Microsoft^®^ Excel, by calculating Pearson’s product–moment (*r*) and its probability (*P*). The correlation analysis was run in pairs of genotypes from the same starch group including the genotypes’ mean values of all developmental stages. Significant correlations (*P* < 0.05) were visualized in matrices using Multi Experiment Viewer software version 4.8.1 (Saeed *et al.*, 2003). The large number of correlations tested may increase false-positive rates, but correction for an inflated Type-1 error may cause a reduction in power due to the small sample size (*n*=10). Therefore, heat maps show all correlations whose unadjusted *P*-value is <0.05, also allowing a fair comparison with literature data (Carrari *et al.*, 2006; Fait *et al.*, 2008; Lombardo *et al.*, 2011) where no adjustment for multiple testing was carried out. Non-corrected and false discovery rate-corrected *r* critical values are shown in Supplementary Table S8 at *JXB* online.

Transcript data (Supplementary Fig. S3 at *JXB* online) were analysed with physical measurements (FW, DW, DM; 2007 HY), growth indexes [FW relative growth rate (RGR) and DW RGR], and metabolites [starch, Fru, Glc, Suc, *myo*-inositol, galactose (Gal); 2005 HY] by a redundancy analysis (RDA), a principal component analysis (PCA) of multivariate regression predicted values that integrates independent data sets (Stewart and Love, 1968). RDA plots are interpreted in the same way as a PCA (Zuur *et al.*, 2007). The RDA was chosen to account for samples collected in different years. RGRs were calculated as described in Moscatello *et al.* (2011).

## Results

### *Metabolite profiling in* A. deliciosa *genotypes during fruit development*

Fifty-one metabolites were identified by GC-TOF-MS (Fig. 1) and subdivided into nine groups: sugars (A), sugar-alcohols (B), sugar phosphates (C), other sugars (D), other metabolites (E), amino acids (F), non-protein amino acids (G), organic acids in the tricarboxylic acid (TCA) cycle (H), and other organic acids (I).

Suc, Fru, Glc, and *myo*-inositol were the major soluble sugars identified by GC-TOF-MS (Fig. 1A, B), and quantified by IC (Supplementary Fig. S1E–H at *JXB* online), whereas all the other sugars detected had lower concentrations. Planteose, a major sugar in kiwifruit leaves (Klages *et al.*, 2004), was also detected in fruit tissues (Fig. 1A). Starch and sugar concentrations declined during cell division and then increased again during cell expansion (Fig. 1A, B, E), while Glc peaked during cell division prior to net starch accumulation. Trehalose (Tre) had a Glc-like pattern. Rhamnose (Rha), melibiose, erythritol, and Gol had starch-like patterns (Fig. 1A, B, E). High-starch genotypes (G3 and G30) had significantly more Suc, Rha, erythritol, Gol, glucosamine, and quinate-3-caffeoyl than low-starch genotypes (G25 and G17) (Fig. 1A, B, D, E). In contrast, low-starch genotypes had significantly more Tre, xylose (Xyl), and α-methyl-glucoside than high-starch genotypes (Fig. 1A, D).

Glutamate, asparagine, glutamine, and γ-aminobutyric acid (GABA) decreased during cell expansion, whereas aspartate increased (Fig. 1F, H). Malate and citrate increased in concentration during cell expansion (Fig. 1G). GABA followed a Glc-like pattern (Fig. 1H) whereas aspartate, arginine, tryptophan, and citrate showed starch-like patterns (Fig. 1F, G). Quinate decreased throughout fruit development (Fig. 1I). Tryptophan, arginine, aspartate, glycine, and glutamine were significantly higher in high-starch genotypes than in low-starch genotypes, as were serine and asparagine, but generally only at cell division (Fig. 1F).

### Carbohydrate and amino acid enzymes during fruit development

**Fig. 2.**
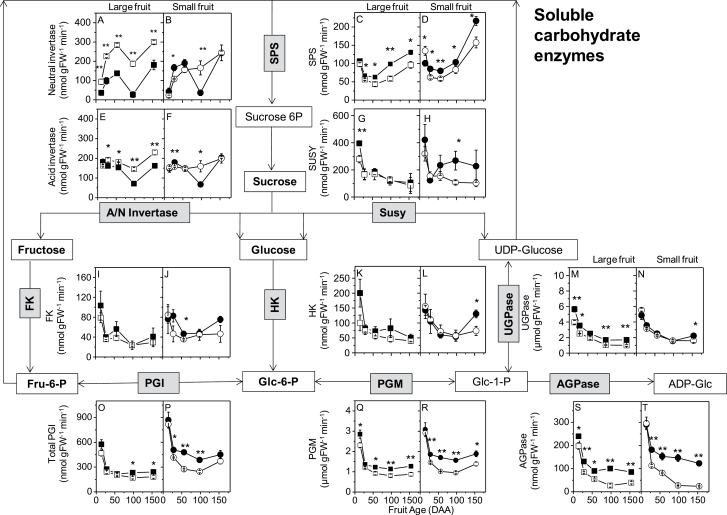
Kiwifruit soluble enzyme activities associated with Suc to starch metabolism. The graph for each enzyme activity is placed in proximity to the enzyme in the simplified pathway chart: A–B, neutral invertase; C–D, sucrose phosphate synthase; E–F, acid invertase; G–H, sucrose synthase; I–J, fructokinse; K–L, hexokinase; M–N, UDP-Glc pyrophosphorylase; O–P, total phosphoglucose isomerase; Q–R, phosphoglucomutase; S–T, ADP-Glc pyrophosphorylase. *t*-test significance levels are reported: *P < 0.05; ** *P* < 0.01. Where the symbol is missing, the difference is not statistically significant. Four genotypes were examined: G3, large fruit and high starch (filled squares); G25, large fruit and low starch (open squares); G30, small fruit and high starch (filled circles), and G17, small fruit and low starch (open circles). Enzyme codes: AGPase, ADP-Glc pyrophosphorylase; A/N invertase, acid and neutral invertase; FK, fructokinase; HK, hexokinase; PGM, phosphoglucomutase; PGI, phosphoglucose isomerase (total); SPS, sucrose phosphate synthase; SuSy, sucrose synthase; UGPase, UDP-Glc pyrophosphorylase. Metabolite abbreviations: ADP-Glc, ADP-glucose; Fru-6-P, fructose-6-phosphate; Glc-1-P, glucose-1-phosphate; Glc-6-P, glucose-6-phosphate. Fruit age is in days after anthesis (DAA). Values are means of four biological replicates ±SEM. Data refer to 2009 outer pericarp samples. White boxes, metabolites; grey boxes, enzymes; bold text, measured metabolites.

The majority of the selected soluble enzymes of Suc, starch, and amino acid metabolism were cytosolic. NI activity (Fig. 2A, B) had a Glc-like pattern, whilst SPS activity (Fig. 2C, D) followed a similar trend to Suc, starch, and DM. Most of the enzyme activities measured in the Suc and starch biosynthetic pathways were higher at cell division than in later stages: e.g. SUSY (Fig. 2G, H), hexokinase (HK; Fig. 2K, L), UDP-Glc pyrophosphorylase (UGPase; Fig. 2M, N), phosphoglucoisomerase (PGI; Fig. 2O, P), phosphoglucomutase (PGM; Fig. 2Q, R), and AGPase (Fig. 2S, T). SPS was the only sugar biosynthetic enzyme with activity levels consistently higher in high-starch genotypes (G3 and G30) than in low-starch genotypes (G25 and G17) (Fig. 2C, D). Total PGM and the first enzyme of starch synthesis in the plastid, AGPase, were significantly higher in high-starch genotypes than in low-starch genotypes (Fig. 2Q–T).

To test which enzymes may have a key role in fruit starch accumulation, the correlation coefficients were estimated for the enzymes of the Suc to starch pathway: AGPase, 0.23; SUSY, 0.10; PGM, 0.19; PGI, 0.13; UGPase, –0.06; HK, 0.14; and fructokinase (FK), 0.12. Enzymes outside the Suc to starch pathway, but important for carbohydrate dynamics, gave the following coefficients: SPS, 0.50; acid invertase (AI), –0.20; NI, –0.33.

All three amino acid metabolism enzymes measured fell 3-fold during cell division (Supplementary Fig. S2 at *JXB* online). Only shikimate dehydrogenase (SDH) increased again during cell expansion. Both glutamate dehydrogenase (GLDH) and aspartate aminotransferase (AST) activities were higher in high-starch genotypes than in low-starch genotypes.

### Transcript analysis of gene families impacting cytosolic sugar concentrations

The PFR *Actinidia* EST (expressed sequence tag) database (Crowhurst *et al.*, 2008) was mined for genes potentially impacting cytosolic sugar concentrations. Thirty-three genes were identified, many of which belonged to multigene families. *Actinidia* genes were named after the nearest *Arabidopsis* homologue, as identified by phylogenetic analysis (data available on request): α-amylase (*AMY1*, *AMY2*, *AMY3*), AGPase (*APL1*, *APL2*, *APL4*, *APS1*), AI (*INV3*, *CWINV1*), β-amylase (*BAM1*, *BAM2*, *BAM3*, *BAM8*, *BAM9*), Dof zinc finger transcription factor (*DOF2*), FK (*FK4*, *FK6*, *FK8*), hexose transporter (*STP1*, *STP3*, *STP14*), HK (*HK1*, *HK3*, *HKL1*), NI (*INVD*, *INVE*, *INVK*), sucrose non-fermenting kinase (*SnRK1*), sucrose transporter (*SUC3*, *SUC4*), and SUSY (*SUSA*, *SUS1*, *SUS6*).

Based on the TargetP predicted location of the enzymes from transcripts (Emanuelsson *et al.*, 2007) and published literature, *AMY2*, *BAM9*, *SUSA*, *SUS1*, *INK*, *APL1*, *APL2*, *APL4*, *SUC3*, *SUC4*, *STP1*, *STP3*, *STP7*, *INV3*, *FK4*, *FK6*, and *SPS Family A (SPSA)* were cytosolic, *AMY3*, *BAM3*, *APS1*, *DOF2*, and *HKL1* were plastidial, *CWINV1* and *AMY1* were apoplastic, and *HK1* was associated with the mitochondria. A homologue of the tomato *LIN5* cell wall invertase, reported to be critical in phloem Suc unloading (Fridman *et al.*, 2004), is absent from the PFR *Actinidia* EST database. In addition, it was not possible to isolate this isoform from fruit tissue by qPCR and sequencing (data not shown). Kiwifruit cell wall invertase *CWINV1* was only expressed in vegetative tissue (data not shown).

**Fig. 3.**
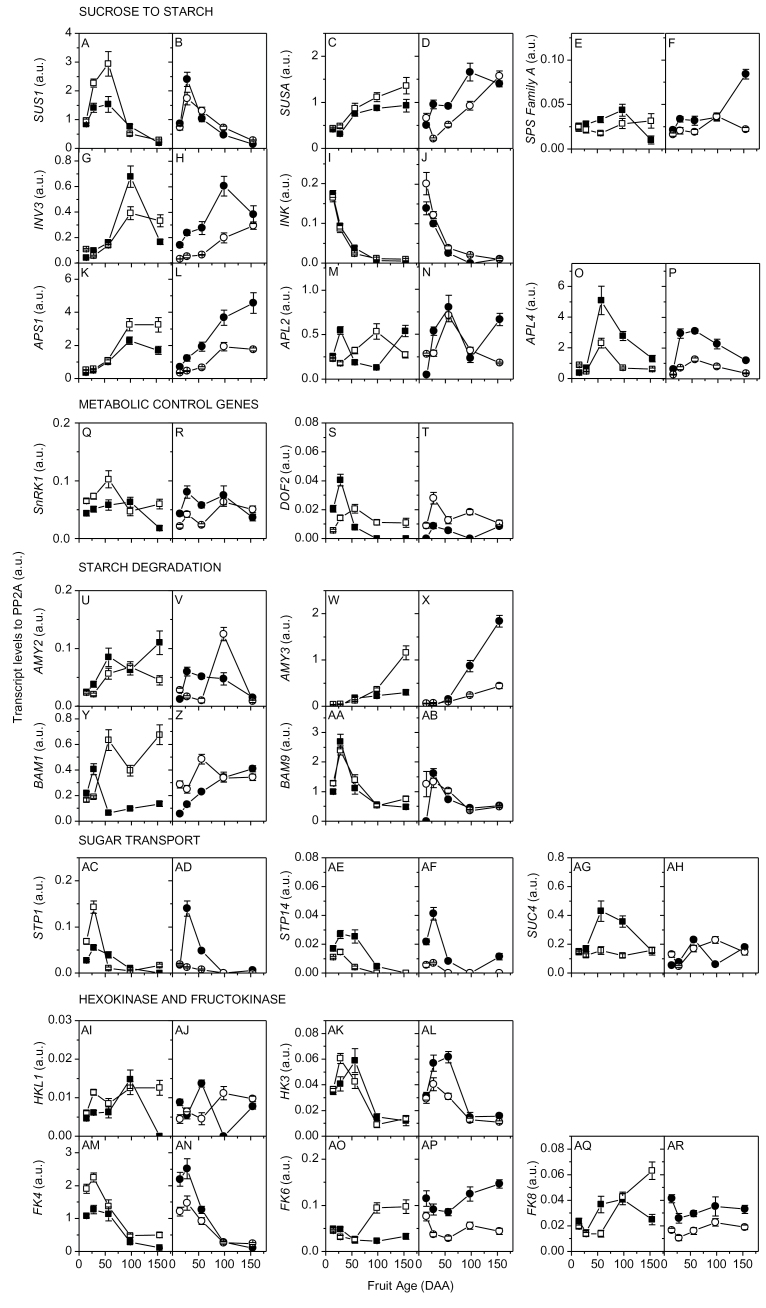
Transcript levels of kiwifruit genes involved in carbohydrate metabolism. Transcript levels measured during fruit development of four *Actinidia deliciosa* genotypes: G3, high starch and large fruit (filled squares); G25, low starch and large fruit (open squares); G30, high starch and small fruit (filled circles); and G17, low starch and small fruit (open circles). Gene codes: sucrose synthase, (A–B) *SUS1*, (C–D) *SUSA*; sucrose phosphate synthase, (E–F) *SPS Family A*; vacuolar invertase, (G–H) INV3; neutral invertase, (I–J) INK; ADP-glucose pyrophosphorylase, (K–L) *APS1*, (M–N) *APL2*, (O–P) *APL4*; sucrose non-fermenting related kinase, (Q–R) *SnRK1*; DOF2 zinc finger transcription factor, (S–T) *DOF2*; α-amylase, (U–V) *AMY2*, (W–X) *AMY3*; β-amylase, (Y–Z) *BAM1*, (AA–AB) *BAM9*; monosaccharide transporters, (AC–AD) *STP1*, (AE–AF) *STP14*; sucrose transporter, (AG–AH) *SUC4*; hexokinase, (AI–AJ) *HKL1*, (AK–AL) *HK3*; fructokinase, (AM–AN) *FK4*, (AO–AP) *FK6*, (AQ–AR) *FK8*. Values are averages of four technical replicates of the reverse-transcribed RNA sample of six pooled biological replicates ±SEM. Housekeeping gene: protein phosphatase 2A (*PP2A*). Fruit age is given in days after anthesis (DAA). Data refer to 2009 outer pericarp samples.

Gene transcription patterns in whole fruit tissue from five high- and five low-starch genotypes (2007 HY; Supplementary Table S2 at *JXB* online) were surveyed during fruit development (Supplementary Fig. S3 at *JXB* online). Genes present in fruit tissues, showing differential transcript abundance between high- and low-starch genotypes, or overall high expression levels, were analysed further. The transcript abundance of 21 genes meeting these criteria (plus *SPSA*) was measured in the same samples used for metabolic and enzyme activity profiling (2009 HY; Fig. 3). *SUS1* (Fig. 3A, B), *APL2* (Fig. 3M, N), *BAM9* (Fig. 3AA, AB), *HK3* (Fig. 3AK, AL), and *FK4* (Fig. 3AM, AN) peaked early in fruit development in a pattern similar to Glc. *SUSA* (Fig. 3C, D), *SPSA* (Fig. 3E, F), *INV3* (Fig. 3G, H), *APS1* (Fig. 3G, H), *AMY3* (Fig. 3U, V), and *FK6* (Fig. 3AO, AP) increased during cell expansion. *APL4* (Fig. 3O, P) and *STP14* (Fig. 3AE, AF) transcripts were higher in high-starch genotypes (G3 and G30) than in low-starch genotypes (G25 and G17) during cell expansion and during cell division, respectively.

### Metabolite, enzyme, and transcript correlations in high- and low-starch genotypes

Correlations were calculated to assess the associations between metabolite, enzyme, and transcript during *A. deliciosa* fruit development. High- (G3 and G30) and low-starch (G25 and G17) genotypes were compared.

Significant metabolite (51 metabolites plus starch, FW, and DM) correlations (*P* < 0.05) are shown in Supplementary Fig. S4 at *JXB* online. For both genotype groups, Suc was positively correlated to DM but not to starch concentration; Tre was positively correlated to Glc; Rha was positively correlated to starch, Gol, Fru, planteose, and DM; planteose was positively correlated to Gal; and starch was positively correlated to Gol. In low-starch genotypes only, starch was positively correlated to Glc and Tre.

Enzyme activities were highly correlated to one another during development in both genotype groups (*P* < 0.05; Supplementary Fig. S5 at *JXB* online). The majority of the correlations were positive, but NI was negatively correlated to 10 other enzymes and positively correlated to AI.

Transcripts only had a limited number of significant correlations (*P* < 0.05; Supplementary Fig. S6 at *JXB* online). For both genotype groups, *SUSA* transcripts were positively correlated to *AMY3*, *INV3*, and *APS1*, as were *FK4* transcripts to *INK*, *SUS1*, *STP1*, *STP14*, and *HK3*. *SUS1* transcripts were positively correlated to *BAM9* and *HK3* in both high- and low-starch genotypes and to *APL4* in high-starch genotypes only. *SUSA* transcripts were negatively correlated to *BAM9* in both genotype groups.

**Fig. 4.**
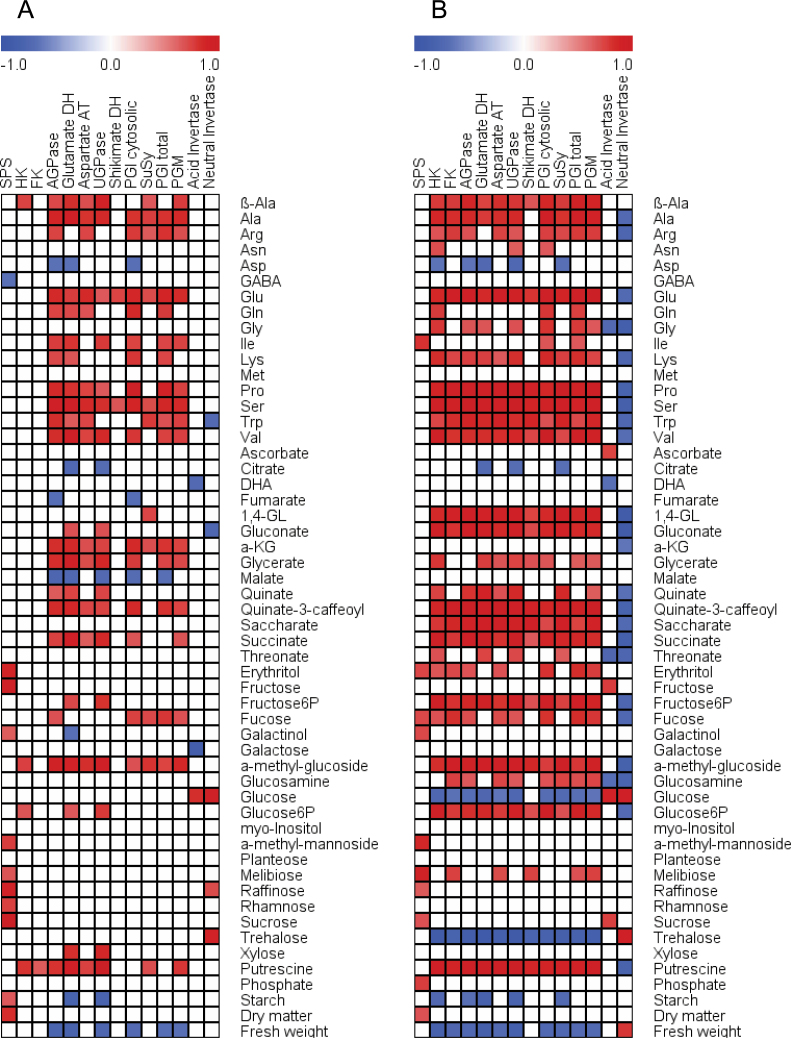
Metabolite to enzyme activity correlation matrix during fruit development of kiwifruit berries. (A) High-starch genotypes (G3 and G30); (B) low-starch genotypes (G25 and G17). The analysis included 51 metabolites analysed by GC-TOF-MS plus starch, fresh weight, and dry matter, and 14 enzymes. The significance threshold of *P* < 0.05 was used (open squares were not statistically significant). 1,4-GL, glucaric acid 1,4-lactone; DHA, dehydroascorbate; α-KG, α-ketoglutarate; AGPase, ADP-glucose pyrophosphorylase; Aspartate AT, aspartate aminotransferase; FK, fructokinase; HK, hexokinase; Glutamate DH, glutamate dehydrogenase; PGM, phosphoglucomutase; PGI, phosphoglucose isomerase; shikimate DH, shikimate dehydrogenase; SPS, sucrose phosphate synthase; SuSy, sucrose synthase; UGPase, UDP-glucose pyrophosphorylase. Data refer to 2009 outer pericarp samples.

Significant metabolite to enzyme correlations (*P* < 0.05) are shown in Fig. 4. SPS was positively correlated to DM in both genotype groups and with Rha in high-starch genotypes. Starch was positively correlated to SPS in high-starch genotypes, and negatively correlated to GLDH and UGPase. In low-starch genotypes, there were only negative correlations between starch and the activities of AGPase, SUSY, UGPase, GLDH, and HK. For correlations between enzymes and their substrate/product, Suc was positively correlated to SPS enzyme activity, Glc6P was positively correlated to HK, and Glc was positively correlated to AI and NI activities for both genotype groups. In low-starch genotypes only, fructose 6-phosphate (Fru6P) was positively correlated to FK, Glc was negatively correlated to HK, AI was positively correlated to Suc and Fru, PGI was positively correlated to Fru6P and glucose 6-phosphate (Glc6P), and PGM was positively correlated to Glc6P. In the amino acid pathway, GLDH was positively correlated to Glc for both genotype classes, whereas it was positively correlated to α-ketoglutarate only in high-starch genotypes. Most of the enzymes correlated negatively to FW.

Significant (*P* < 0.05) metabolite to transcript (Supplementary Fig. S7 at *JXB* online) and transcript to enzyme (Supplementary Fig. S8 at *JXB* online) correlations were scarce, with little consistency between high- and low-starch genotypes and no correlations between genes and their respective enzyme activity.

### Redundancy analysis of transcript levels, and metabolite,and physical measurements

**Fig. 5.**
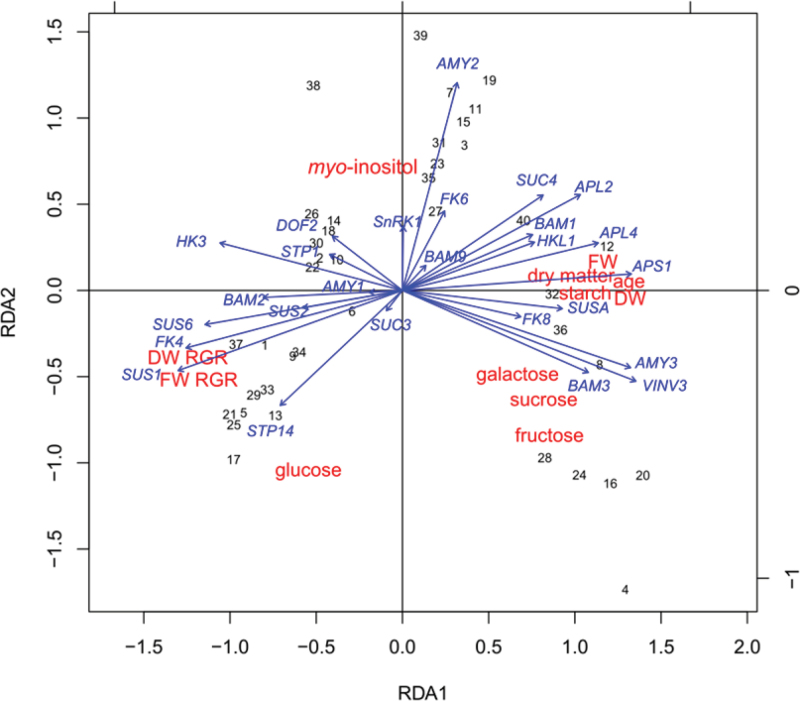
Redundancy analysis of physical measurements, growth rates, metabolites, and transcript levels obtained from kiwifruit genotypes. Fruit samples used in this analysis were collected in 2005 and in 2007 (Supplementary Table S1 at *JXB* online) and were representative of all fruit tissue types. Gene codes: α-amylase (*AMY1*, *AMY2*, *AMY3*), ADP-glucose pyrophosphorylase (*APL2*, *APL4*, *APS1*), β-amylase (*BAM1*, *BAM2*, *BAM3*, *BAM9*), DOF zinc finger (*DOF2*), fructokinase (*FK4*, *FK6*, *FK8*), hexokinase (*HK3*, *HKL1*), sucrose non-fermenting kinase (*SnRK1*), monosaccharide transporter (*STP1*, *STP14*), sucrose transporter (*SUC3*, *SUC4*), sucrose synthase (*SUS1*, *SUS6*, *SUSA*), vacuolar invertase (*INV3*). Other abbreviations: DW, dry weight; FW, fresh weight; RGR, relative growth rate. Numbers represent each genotype/sampling date combination (Supplementary Table S2 at *JXB* online). Colour coding: red, physical measurements and metabolites; blue, gene transcript abundance; black, sample number.

To test if the results of this study were applicable to a larger number of genotypes, the existing data sets for fruit development and carbohydrate composition (Nardozza *et al.*, 2010) were re-analysed by RDA together with transcript abundance (Supplementary Fig. S3 at *JXB* online) measured for 10 genotypes with contrasting starch concentrations (Fig. 5). Starch, DM, and DW were positively correlated with dimension1 (RDA1), whereas FW RGR and DW RGR were negatively correlated with RDA1. *APL4* and *APS1* transcripts were connected to starch and DM, as they were largely explained by RDA1. *SUS1* was connected to both FW RGR and DW RGR, whereas SUSA was connected to starch. *FK4* was connected to *SUS1* and again to both FW RGR and DW RGR.

## Discussion

Kiwifruit metabolism is different from that of the other fruiting species examined to date as carbon is mainly stored as starch. Here it is shown that NI activity is likely to contribute to the rise in Glc concentration associated with cell division, while *BAM9*/*AMY2* transcripts and SPS/SUSY activities are likely to be involved in starch turnover and Suc cycling, respectively, throughout fruit development. Novel compounds in kiwifruit berries, including planteose and Gol, were also identified. Gol, a minor metabolite associated with high-starch genotypes, is possibly involved in planteose metabolism. The innovative approach of comparing kiwifruit genotypes contrasting in fruit size and starch concentration allowed the identification of AGPase as a critical metabolic step that contributed to enhanced starch accumulation in high-starch genotypes. Therefore, the integration of metabolite, enzyme, and transcript data highlights novel aspects of carbon metabolism in kiwifruit that may be transferable to other starch-accumulating fruit.

### Cell division and Glc accumulation

Kiwifruit show important developmental differences from other well studied fruits. At fruit set, the fruit had significant levels of starch and Suc which declined during the first 20–30 DAA. Incoming Suc, together with Suc and starch already present in the fruit at anthesis, are likely to support both cell division and accumulation of Glc. Kiwifruit cell division is characterized by a peak in Glc (Klages *et al.*, 1998; Nardozza *et al.*, 2010) not observed in other fruit as tomato maximize their starch accumulation (Baxter *et al.*, 2005; Nardozza *et al.*, 2010) and peach and melon slowly accumulate soluble carbohydrates during this phase (Dai *et al.*, 2011; Lombardo *et al.*, 2011). In this study, NI activity increased in parallel to the increase in Glc concentration during cell division, presumably to metabolize Suc unloaded during this period. In contrast, hydrolysis of Suc by invertase in other fruit usually occurs during rapid cell expansion, when hexoses double their osmotic contribution (Ruan and Patrick, 1995). The high FK/HK enzyme activities and *FK4/HK3* transcripts early in fruit development were probably involved with the phosphorylation of Fru and Glc released by NI.

*BAM9* (cytosolic) also peaked at cell division and was highly transcribed (Fig. 3AA, AB). In addition *AMY2* (cytosolic) transcripts was present throughout fruit development (Fig. 4K, L). It is suggested that these amylases are involved in cytosolic starch or carbon turnover during fruit development. In *Arabidopsis* leaves, an involvement of *BAM9* and *AMY2* in the degradation of unknown cytosolic glucans was proposed (Smith *et al.*, 2004). *BAM9* may also interact with heteroglycans recently demonstrated to participate in starch turnover in potato (*Solanum tuberosum* L.) and *Arabidopsis* (Fettke *et al.*, 2009).

### Cell expansion and starch accumulation

From ~50 DAA as the fruit cells expanded, kiwifruit accumulated DM predominantly as starch (Boldingh *et al.*, 2000; Nardozza *et al.*, 2010), unlike tomato, where soluble sugars accumulated (Baxter *et al.*, 2005; Nardozza *et al.*, 2010). During this period, characterized by a decrease in Glc concentration and NI activity, starch, Suc, and SPS activity increased. The drop in Glc, a feature not apparent in other fruits, together with the decrease in GABA and Tre, may be signals for cell expansion and starch accumulation. Although most of the soluble metabolites and enzymes are generally diluted by growth (Figs 1, 2), malate and citrate increased significantly, confirming their involvement in the osmotic regulation of fruit expansion (Nardozza *et al.*, 2010). *APL4* transcripts coding for an AGPase large subunit did not increase as starch accumulation increased, suggesting that sufficient enzyme, synthesized perhaps in response to the Glc peak, was made available earlier to support full starch accumulation.

At harvest (154 DAA), kiwifruit are considered ‘mature’ but unripe (MacRae *et al*., 1989, 1992). A number of metabolic changes were associated with fruit approaching maturity: starch concentrations reached their maximum or plateaued (Supplementary Fig. S1E at *JXB* online), Suc and hexoses started to accumulate (Fig. 1A), and SPS activity (Fig. 2C, D) and *AMY3* transcripts (Fig. 3W, X; Supplementary Fig. S3 at *JXB* online) increased significantly. As *AMY3* is the only plastidial α-amylase (Streb and Zeeman, 2012), its increased transcription could indicate that fruit are mature with increased cycling between sugars in the cytosol and starch in the plastid, or, alternatively, the readiness of the fruit to use its starch to progress ripening. Unexpectedly, *BAM1* transcripts, a plastidial β-amylase critical in leaf starch degradation (Streb and Zeeman, 2012), did not increase along with *AMY3*. However, *BAM3* (also plastidial) could be a candidate for starch degradation in heterotrophic tissues as its transcripts significantly increased by 154 DAA (Supplementary Fig. S3 at *JXB* online) and were strongly up-regulated in ripening kiwifruit (Atkinson *et al.*, 2011; Richardson *et al.*, 2011).

While NI and AI activity and Glc increased at maturity, SUSY activity and *SUS1* transcripts declined during starch accumulation and remained low in mature fruit, suggesting a more critical role for invertase in kiwifruit metabolism. In contrast, *SUSA* increased with the rise in starch, Suc, and SPS activity, becoming the dominant SUSY in mature fruit, as previously reported in kiwifruit (Richardson *et al.*, 2004), melon (Dai *et al.*, 2011), and citrus (Komatsu *et al.*, 2002). These data, together with the strong positive correlation between *SUSA*, starch, and DM in the RDA (Fig. 5), suggest the involvement of *SUSA* in Suc cycling in the fruit cell rather than fruit Suc unloading.

### Correlations between metabolites, enzyme activity, and transcripts

Metabolism profiling of *A. deliciosa* fruit showing extremes of starch accumulation revealed correlations between metabolites, enzyme activity, and transcripts. The positive and significant correlation between SPS activity and Suc (Fig. 4) suggests a role for SPS in Suc cycling. *SPSA* transcripts were also correlated to Suc, but only in high-starch genotypes (Supplementary Fig. S7A at *JXB* online). There have been various reports of correlations between SPS enzyme activity and Suc (Dai *et al.*, 2011), or transcript levels and Suc (Xue *et al.*, 2013). Interestingly, the *Arabidopsis SPSA1* null mutant also demonstrated strong correlations between starch, Suc, and enzyme/transcript levels (Sun *et al.*, 2011).

Metabolite to transcript or enzyme to transcript correlations (Supplementary Figs S7, S8 at *JXB* online) were generally infrequent, and no correlations between transcript levels and their respective enzyme activities were observed. Previous studies of metabolism profiling in other fruit or systems (Gibon *et al.*, 2004; Steinhauser *et al.*, 2010; Dai *et al.*, 2011; Lombardo *et al.*, 2011) have shown that metabolites, enzyme activities, and transcripts are often interconnected but not in a simple way. For example, a transcriptional response does not necessarily lead to a functional response so metabolites often appear more coordinated than transcripts. In particular, transcripts do not usually correlate to functionally related enzymes or metabolites (Gibon *et al.*, 2004; Piques *et al.*, 2009; Steinhauser *et al.*, 2010; Dai *et al.*, 2011); as enzyme assays do not distinguish between isoforms of multigene families, enzyme turnover is slower than transcript turnover or an enzyme might be modified post-translationally (Gibon *et al.*, 2004; Carrari *et al.*, 2006; Piques *et al.*, 2009; Steinhauser *et al.*, 2010; Dai *et al.*, 2011).

### Sugar unloading in the fruit

The present data suggest that kiwifruit phloem unloading may be symplastic throughout fruit development as cytosolic enzymes, NI and SUSY (Fig. 2A, B, G, H), were the dominant Suc-cleaving enzymes. The presence of symplasmic connectivity between phloem cells and vascular parenchyma cells (Gould *et al.*, 2013) and the lack of both cell wall invertase activity (Moscatello *et al.*, 2011) and the gene for a fruit-specific cell wall invertase (this study) in kiwifruit berries support this hypothesis. Tomato phloem unloading is still debated, and evidence supporting two contrasting theories has been produced using different systems. The introgression of a cell wall invertase exotic allele (*LIN5*) into cultivated tomato showed its critical role for Suc apoplastic unloading (Fridman *et al.*, 2004; Zanor *et al.*, 2009) and fruit starch accumulation (Baxter *et al.*, 2005) early in fruit development. Conversely, elevated SUSY activity at the time of high Suc unloading was associated with Suc symplastic unloading early in fruit development (Nguyen-Quoc *et al.*, 1999; Nguyen-Quoc and Foyer, 2001). Similarly to kiwifruit, transcript and biochemical studies also failed to detect the *LIN5* homologue fruit cell wall invertase in melon and peach fruit (Dai *et al.*, 2011; Lombardo *et al.*, 2011).

The trisaccharide planteose, a raffinose oligosaccharide (RFO) family sugar, was found by GC-TOF-MS (Fig. 1A) and quantified in trace amounts by IC in kiwifruit flesh. Planteose was first identified in *A. arguta* as a short-term storage form of Suc in leaves and as a transport sugar in phloem exudates (Klages *et al.*, 2004), but it has not been previously reported in fruit. *myo*-Inositol, a common *Actinidia* fruit sugar-alcohol, is a precursor for the synthesis of raffinose: first UDP-Gal and *myo*-inositol form Gol, then Suc and Gol form raffinose (Sprenger and Keller, 2000). In this study, both *myo*-inositol and Gol were present in *A. deliciosa* fruit, as well as raffinose and planteose (Fig 1A, B). Following a possible symplastic transfer of planteose to the fruit (Klages *et al.*, 2004), planteose is broken down to the trace levels measured herein, thereby releasing Gal which may return to storage as Gol or enter the Glc pool, possibly in a similar way to raffinose (Dai *et al.*, 2011). So far kiwifruit is the only fleshy fruit species where planteose has been identified, and planteose metabolism represents a novel research area as neither its synthetic nor its degradation pathway within kiwifruit is known, and its phloem loading and unloading mechanisms have not been characterized. Finally, a link between RFO sugar translocation and the lack of cell wall invertase is suggested, as these two conditions also characterize melon fruit (Schaffer *et al.*, 1996; Dai *et al.*, 2011).

### What are the causes of variation in starch concentration?

Comparison of *A. deliciosa* high-starch genotypes (G3 and G30) with low-starch genotypes (G25 and G17) during fruit development shows that higher rates of starch accumulation are consistently associated with higher final starch concentrations, despite differences in fruit size. The 2- to 5-fold higher AGPase activity found in high-starch genotypes, which was also maintained for a longer period of time, was a determinant of high starch concentration (Fig. 2S, T). High-starch kiwifruit genotypes also had at least 2-fold higher levels of *APL4* transcripts (Fig. 3O, P). AGPase is the first committed step in the starch synthesis pathway (Preiss *et al.*, 1987), synthesizing ADPGlc from the precursor Glc1P—originating in the cytosol from cytoplasmic sugar metabolism. The AGPase involvement in starch metabolism was also confirmed in a wider selection of genotypes by the correlation between the AGPase isoforms (*APL4* and *APS1*) and starch/DM (Fig. 5). In near-isogenic lines of tomato, the high-starch phenotype was linked to a temporal extension of transcription of an AGPase large subunit gene (*AgpL*) that also conferred higher AGPase activity to the high-starch tomato line (Petreikov *et al.*, 2006). AGPase may therefore be the enzyme contributing to the starch concentration variations observed in outer pericarp small cells of these *A. deliciosa* genotypes (Nardozza *et al.*, 2011*b*).

SPS activity was also significantly higher in high-starch genotypes throughout fruit development (Fig. 2C, D), and the correlation coefficient suggested that the SPS contribution in starch synthesis was substantial (0.50). SPS has been associated with increased starch in other plants. Kerr *et al.* (1984) observed positive correlations between SPS activity and DM accumulation in soybean [*Glycine max* (L.) Merr.], whereas the overexpression of a maize SPS gene in tomato fruit increased starch accumulation (Nguyen-Quoc *et al.*, 1999).

Gol curves followed a starch-like pattern and its concentration was higher in high-starch genotypes compared with low-starch genotypes (Fig. 1B). Lombardo *et al.* (2011) suggested that peach mesocarp Gol levels were involved in seed formation. In kiwifruit, the high outer pericarp Gol levels of high-starch genotypes could be associated with the higher numbers of seeds present in the inner pericarp of these genotypes (Nardozza *et al.*, 2010).

### Conclusion

Metabolic profiling in kiwifruit, a starch-storing fruit, has shown that *A. deliciosa* is a metabolic variant compared with other well studied fruit. This study identifies some features leading to high-starch kiwifruit and already evident at fruit set, for example AGPase and SPS activities, and Suc and starch concentrations. Kiwifruit accumulates Glc at cell division and its decline coincided with a loss of NI and the accumulation of starch, citrate, and malate. *APL4* transcription and AGPase activity, together with SPS activity, have a role in the level of starch accumulation, as evidenced by the contrast between high- and low-starch-accumulating genotypes. This study is relevant for the genetic improvement of quality and flavour in kiwifruit as achieving high starch concentration, hence high DM, is important for consumer acceptance (Harker *et al.*, 2009; Nardozza *et al.*, 2011*a*), while planteose and Gol metabolism in kiwifruit represent novel research areas.

## Supplementary data

Supplementary data are available at *JXB* online.

Figure S1. Changes in starch, physical measurements, and ion chromatography quantified soluble sugars and the sugar-alcohol *myo*-inositol in outer pericarp during fruit development *of Actinidia deliciosa* genotypes (2009 samples).

Figure S2. Kiwifruit soluble enzyme activities associated with amino acid metabolism (2009 samples).

Figure S3. Heat map of fruit developmental transcript variations in 10 *Actinidia deliciosa* genotypes for a selection of carbohydrate metabolism genes (2007 samples).

Figure S4. Metabolite correlations in fruit outer pericarp of *Actinidia deliciosa* genotypes (2009 samples).

Figure S5. Enzyme activity correlations in fruit outer pericarp of *Actinidia deliciosa* genotypes (2009 samples).

Figure S6. Transcript correlations in fruit outer pericarp of *Actinidia deliciosa* genotypes (2009 samples).

Figure S7. Hierarchical cluster analysis of metabolite to transcript correlations in fruit outer pericarp of *Actinidia deliciosa* genotypes (2009 samples).

Figure S8. Transcript to enzyme correlations in fruit outer pericarp of *Actinidia deliciosa* genotypes (2009 samples).

Table S1. List of *Actinidia deliciosa* fruit sample types involved in the present study.

Table S2. List of *Actinidia deliciosa* fruit samples included in the redundancy analysis.

Table S3. *Actinidia deliciosa* fruit developmental stages sampled in 2009 harvest year, with relative BBCH stage and description.

Table S4. Enzyme extraction buffer composition.

Table S5. Dilution of extraction buffer to tissue fresh weight used for each kiwifruit enzymatic assay.

Table S6. Methods adopted for enzyme activity assays.

Table S7. List of primers used for transcript abundance analysis, with respective GenBank identification.

Table S8. Effects on correlation analyses of adjustment of critical significance thresholds for Pearson’s correlation coefficients to control the false discovery rate.

Supplementary Data

## Abbreviations:

AGPase: ADP-glucose pyrophosphorylase
AI: acid invertase
AST: aspartate aminotransfarase
DAA: days after anthesis
DM: dry matter
DW: dry weight
FK: fructokinase
Fru6P: fructose 6-phosphate
FW: fresh weight
G3: genotype 3
G17: genotype 17
G25: genotype 25
G30: genotype 30
GABA: γ-aminobutyric acid
GC-TOF-MS: gas chromatography-time of flight-mass spectrometry
Glc6P: glucose 6-phosphate
GLDH: glutamate dehydrogenase
Gol: galactinol
HK: hexokinase
NI: neutral invertase
PFR: Plant & Food Research
PGI: phosphoglucoisomerase
PGM: phosphoglucomutase
RDA: redundancy analysis
RGR: relative growth rate
SDH: shikimate dehydrogenase
SPS: sucrose phosphate synthase
SUSY: sucrose synthase
TCA: tricarboxylic acid
Tre: trehalose
UGPase: UDP-glucose pyrophosphorylase.
