# Supplementary material for: Metabolic analysis of kiwifruit (Actinidia deliciosa) berries from extreme genotypes reveals hallmarks for fruit starch metabolism
Source: J Exp Bot. 2013 Sep 21;64(16):5049–63. doi: 10.1093/jxb/ert293 (PMC3830485; doi:10.1093/jxb/ert293)
Supplement: Supplementary Data [file supp_64_16_5049__index.html]

Metabolic analysis of kiwifruit (Actinidia deliciosa) berries from extreme genotypes reveals hallmarks for fruit starch metabolism — Metabolic analysis of kiwifruit (Actinidia deliciosa) berries from extreme genotypes reveals hallmarks for fruit starch metabolism — Supplementary Data 

# Metabolic analysis of kiwifruit (*Actinidia deliciosa*) berries from extreme genotypes reveals hallmarks for fruit starch metabolism

## Supplementary Data

Data files

**Files in this Data Supplement:**

- Supplementary Data - Supplementary Data
