# Supplementary material for: Metabolic analysis of kiwifruit (Actinidia deliciosa) berries from extreme genotypes reveals hallmarks for fruit starch metabolism
Source: J Exp Bot. 2013 Sep 21;64(16):5049–63. doi: 10.1093/jxb/ert293 (PMC3830485; doi:10.1093/jxb/ert293)
Supplement: Supplementary Data [file supp_ert293_jexbot102772_file001.pdf]

Metabolic analysis of kiwifruit (*Actinidia deliciosa*) berries from extreme genotypes reveals hallmarks for fruit starch metabolism

Simona Nardozza, Helen L. Boldingh, Sonia Osorio, Melanie Höhne, Mark Wohlers, Andrew P. Gleave, Elspeth A. MacRae, Annette C. Richardson, Ross G. Atkinson, Ronan Sulpice, Alisdair R. Fernie, Michael J. Clearwater

Supplementary Data

SUPPLEMENTARY TABLES

Supplementary Table S1. List of *Actinidia deliciosa* fruit sample types involved in the present study. CHO, carbohydrates; E, enzyme activities; HY, harvest year; M, metabolites; PM, physical measurements; T, transcripts. Bold font: genotypes more deeply characterised in 2009. *, data sourced from Nardozza et al. (2010).

Genotype ID	Starch class	Size class	Year of selection	SAMPLING								
				2005 HY		2007 HY		2009 HY				
				PM*	CHO*	PM*	T	PM	CHO	M	E	T
1	Low	Large	2003	✓	✓	✓	✓					
**3**	High	Large	2003	✓	✓	✓	✓	✓	✓	✓	✓	✓
5	High	Large	2003	✓	✓	✓	✓					
**17**	Low	Small	2003	✓	✓	✓	✓	✓	✓	✓	✓	✓
**25**	Low	Large	2004	✓	✓	✓	✓	✓	✓	✓	✓	✓
26	Low	Large	2004	✓	✓	✓	✓					
27	Low	Small	2004	✓	✓	✓	✓					
28	High	Large	2004	✓	✓	✓	✓					
29	High	Small	2004	✓	✓	✓	✓					
**30**	High	Small	2004	✓	✓	✓	✓	✓	✓	✓	✓	✓

Supplementary Table S2. List of *Actinidia deliciosa* fruit samples included in the redundancy analysis. Ten genotypes were considered (five high-starch, sample 1 to 20, and five low-starch, sample 21 to 40), and whole fruit tissue was collected in 2007 at four different fruit ages. DAA, days after anthesis.

sample number	genotype	fruit age (DAA)
1	3	28
2	3	56
3	3	98
4	3	154
5	5	28
6	5	56
7	5	98
8	5	154
9	28	28
10	28	56
11	28	98
12	28	154
13	29	28
14	29	56
15	29	98
16	29	154
17	30	28
18	30	56
19	30	98
20	30	154
21	1	28
22	1	56
23	1	98
24	1	154
25	17	28
26	17	56
27	17	98
28	17	154
29	25	28
30	25	56
31	25	98
32	25	154
33	26	28
34	26	56
35	26	98
36	26	154
37	27	28
38	27	56
39	27	98
40	27	154

Supplementary Table S3. *Actinidia deliciosa* fruit developmental stages sampled in 2009 harvest year, with relative Biologische Bundesantalt Bundessortenamt und Chemische Industrie (BBCH) scale stage and description (Richardson *et al.*, 2011). DAA, days after anthesis.

Fruit age (DAA)	BBCH stage	Description
14	70	Fruit set
28	72	Fruit reached 20% of final weight
56	75	50% fruit growth; growth slowing, beginning of net starch accumulation
98	78	80% fruit growth; net starch accumulation
154	80	Fruit mature, but unripe

Supplementary Table S4. Enzyme extraction buffer composition.

Compound	Final concentration
Hepes/KOH, pH 7.5	250 mM
$MgCl_2$	50 mM
EDTA	5 mM
EGTA	5 mM
Benzamidine	5 mM
ε-aminocapronic acid	5 mM
BSA	1.25% (w/v)
Phenylmethylsulfonyl fluoride	1 mM
Leupeptin	20 µM
DTT	0.5 mM
Glycerol	20% (v/v)
Triton X-100	1% (v/v)

Supplementary Table S5. Dilution of extraction buffer to tissue fresh weight (FW) used for each kiwifruit enzymatic assay.

Enzyme	Optimal dilution (Extraction buffer:FW)
AGPase	250
Acid Invertase	1000
Alanine AT	5000
Aspartate AT	5000
FK	500
HK	500
Glutamate DH	500
Neutral Invertase	500
PGM	250
PGI cytosolic	250
PGI total	250
Shikimate DH	250
SPS	250
SUSY	2000
UGPase	2000

Supplementary Table S6. Methods adopted for enzyme activity assays (Gibon *et al.*, 2004; Steinhauser *et al.*, 2010). DHAP, dihydroxyacetone phosphate.

Enzyme	Abbreviation	Type of assay	Assay direction for bidirectional enzymes	Product determination	Notes	Reference
Acid invertase	AI	stopped assay		fluorimetry after the addition of Amplex RedTM (dihydroxyphenoxazine)	same method as NI but buffer pH was 5	Gibon *et al* . (2004)
ADPGlucose pyrophosphorylase	AGPase	stopped assay	via Glc-1-P formation	via a glycerol3P/DHAP cycling reaction		Gibon *et al* . (2004)
Aspartate aminotransferase	AST	stopped assay	via oxalacetate formation	via a NAD/NADH cycling reaction		Gibon *et al* . (2004)
Fructokinase	FK	stopped assay		via a NADP/NADPH cycling reaction		Gibon *et al* . (2004)
Glutamate dehydrogenase	GLDH	stopped assay	via Glu formation	via a NAD/NADH cycling reaction		Gibon *et al.* (2004)
Hexokinase	HK	stopped assay		via a NADP/NADPH cycling reaction		Gibon *et al* . (2004)
Neutral invertase	NI	stopped assay		fluorimetry after the addition of Amplex RedTM (dihydroxyphenoxazine)	same method as AI but buffer pH was 7	Gibon *et al* . (2004)
Phoshoglucose isomerase	PGI	continuous assay	via Glc-6-P formation		Total and cytosolic PGI were measured. Cytosolic PGI was assayed after heating the extract at 50°C for 15 min	Steinhauser *et al.* (2010)
Phosphoglucomutase	PGM	continuous assay	via Glc-6-P formation		Only total PGM was measured.	Steinhauser *et al.* (2010)
Shikimate dehydrogenase	SDH	stopped assay	via 3-dehydroshikimate formation	via a NADP/NADPH cycling reaction		Gibon *et al* . (2004)
Sucrose phosphate synthase	SPS	stopped assay		via a glycerol3P/DHAP cycling reaction		Gibon *et al* . (2004)
Sucrose synthase	SUSY	stopped assay	via UDP-Glc formation	via a NADP/NADPH cycling reaction		Gibon *et al* . (2004)
UDPGlucose pyrophosphorylase	UGPase	stopped assay	via Glc-1-P formation	via a glycerol3P/DHAP cycling reaction		Gibon *et al* . (2004)

Supplementary Table S7. List of primers used for transcript abundance analysis, with respective GenBank (GB) identification (ID). EF1α and PP2A were used as housekeeping genes. All sequences reported in this paper have been submitted to GenBank. *SPSA* primer as per Fung et al. (2003). NA, not available.

GENE	ENZYME FAMILY	GB ID	EST	PRIMER CODE	FORWARD	REVERSE
AMY1	α-Amylase	JX067525	1475696	SN61+62	GAATTGGCTCAAGTCCGAAA	GGACGTGTTTTGCATGTAGTTC
AMY2	α-Amylase	JX067526	1475697	SN63+64	AGGGAATTCTTCAGGAAGCTG	CAAGAAAAGTGACAGCCCTTG
AMY3	α-Amylase	FG460641	244905	SN65+66	AATCACCGCTGTGCACAATA	CCTTCCCTGGAAATGTGGAT
APL2	ADPglucose Pyrophosphorylase	FG456809	189962	SN45+46	AAACAGCCTCCGAAGTTTGA	TTATGGCATCGACAATCCTG
APL4	ADPglucose Pyrophosphorylase	FG521835	311362	SN47+48	GTCATCGTGAACAAAGATGGTG	TGATTGTGGCCTTCTCCAAT
APS1	ADPglucose Pyrophosphorylase	FG458669	192438	SN39+40	TGAGGGTGCAAGCTTACTTGT	AATCTGGCACTGGCTTTTTG
BAM1	β-Amylase	KC171741	435565	SN83+84	GAGACACCATTGTGGAAATTCA	CACTGAAAGGCTCCAATTCC
BAM2	β-Amylase	FG410711	234964	SN69+70	GCAGGTTCTTTCTCAATTGGT	GATAGCTTCACAGCAATGCAA
BAM3	β-Amylase	FG455287	239356	SN71+72	ACGACAAGTACATGAGGGCTTC	TTGGTTATACTGGCCGGAGT
BAM9	β-Amylase	FG460922	197517	SN73+74	TGATCTTCCTCTTCTCGATGG	TGATATGCCCGTGATAGTGG
DOF2	DOF Transcription Factor	JX067534	324501	SN105+106	GTCAACCAGCCCCGATATTT	CGAGCTTTTGTGCTTTCGAC
EF1α	Elongation Factor 1-α	FG418280	21826	SN125+126	GCACTGTCATTGATGCTCCT	CCAGCTTCAAAACCACCAGT
FK4	Fructokinase	FG521968	311495	SN99+100	CCAAGAGCTTTCATGGATCTG	TCCACCATCTTGCACAAAAG
FK6	Fructokinase	FG434733	78318	SN101+102	CTTATCAAGCAGGCGAGCAT	GAAAGGACACAACCGCATTC
FK8	Fructokinase	FG514153	323025	SN103+104	AGAGGTGGCTTGAGAACGAA	AAACATCTTTGCCTCCCTCA
HK3	Hexokinase	FG521281	310807	SN107+108	CCTGGGTGAAATTGTAAGACG	ATCTGGGGTCCTGAGAACAA
HKL1	Hexokinase	JX067540	945716	SN89+90	TGATAATTGGGACGGGTACAA	TGTTAATTACCATGCCTCCAGA
INK	Neutral Invertase	JX067549	583622	SN41+42	TCGACACATTCCCGACTCTT	GCATCTCAAAGCCATGAAGAA
INV3	Vacuolar Invertase	FG438458	101593	SN13+14	TTCGATTCCAATGGTGTGTG	GGTTTTGCACTTGCACGTAA
PP2A	Protein Phosphatase 2A	FG522516	312205	SN25+26	GCAGCACATAATTCCACAGG	TTTCTGAGCCCATAACAGGAG
SnRK1	Sucrose non-Fermenting Kinase	JX067541	945651	SN75+76	TTCAGTCTCAAGCCCATCCT	ACCCACCTGCATTTCATGTT
SPSA	Sucrose Phosphate Synthase	AF318949	NA	Fung et al. (2003)	GAACTTAAAGTTTTCACTGGATG	CGGAGAGATCCGATATAGCACC
SPT1	Hexose Transporter	JX067542	1475738	SN93+94	ACGTTGGTTTCGATTTACGG	CAGGCTGCAACTACAATCTGAC
STP14	Hexose Transporter	FG528269	320319	SN95+96	GCTAGTTGTGGCAGCTTTGA	CCACCGTTACATTGCACATT
SUC3	Sucrose Transporter	JX067543	583663	SN77+78	CTCTTACCTGGTTGTCCTGGTT	ACTTGAGAGACATCCCCTTTTG
SUC4	Sucrose Transporter	JX067544	583617	SN79+80	CAAGGCTTATCGATGGGTGT	GCGAGTTACCACCACCAAAT
SUS1	Sucrose Synthase	FG404527	285852	SN1+2	ACCACTTTTCGTGCCAGTTC	GTCCAACGGTGTTCTTGCTT
SUS6	Sucrose Synthase	KC171739	583619	SN81+82	GAAGCAAGAATAGGCCTGGA	CGGGAGCAGCGATATTAAAC
SUSA	Sucrose synthase	FG439911	100046	SN3+4	CTGCCGAATTACAGGGTGTT	GAGCAATGGTGCACTGTGTT

Supplementary Table S8. Effects on correlation analyses of adjustment of critical significance thresholds for Pearson's correlation coefficients to control the False Discovery Rate (FDR). FDR critical values are calculated for each correlation matrix presented in the manuscript.

	**r values for *p* <0.05**											
	High starch Metabolite-Metabolite correlation Fig. 5A	Low starch Metabolite-Metabolite correlation Fig. 5B	High starch Enzyme-Enzyme correlation Fig. 6A	Low starch Enzyme-Enzyme correlation Fig. 6B	High starch Metabolite-Enzyme correlation Fig. 7A	Low starch Metabolite-Enzyme correlation Fig. 7B	High starch Transcripts-Transcripts correlation Fig. S15A	Low starch Transcripts-Transcripts correlation Fig. S15B	High starch Metabolite-Transcripts correlation Fig. S16A	Low starch Metabolite-Transcript correlation Fig. S16B	High starch Enzyme-Transcripts correlation Fig. S17A	Low starch Enzyme-Transcripts correlation Fig. S17B
Non-corrected critical value	0.64	0.64	0.64	0.64	0.64	0.64	0.64	0.64	0.64	0.64	0.64	0.64
FDR critical value	0.82	0.79	0.70	0.74	0.82	0.72	0.88	0.83	0.94	0.92	n.s.	n.s.
Number of significant correlations	101	193	43	66	54	260	3	9	1	3	0	0
Number of positive significant correlations	91	146	43	56	53	216	2	9	1	1	0	0
Number of negative significant correlations	10	47	0	10	1	44	1	0	0	2	0	0

SUPPLEMENTARY FIGURES

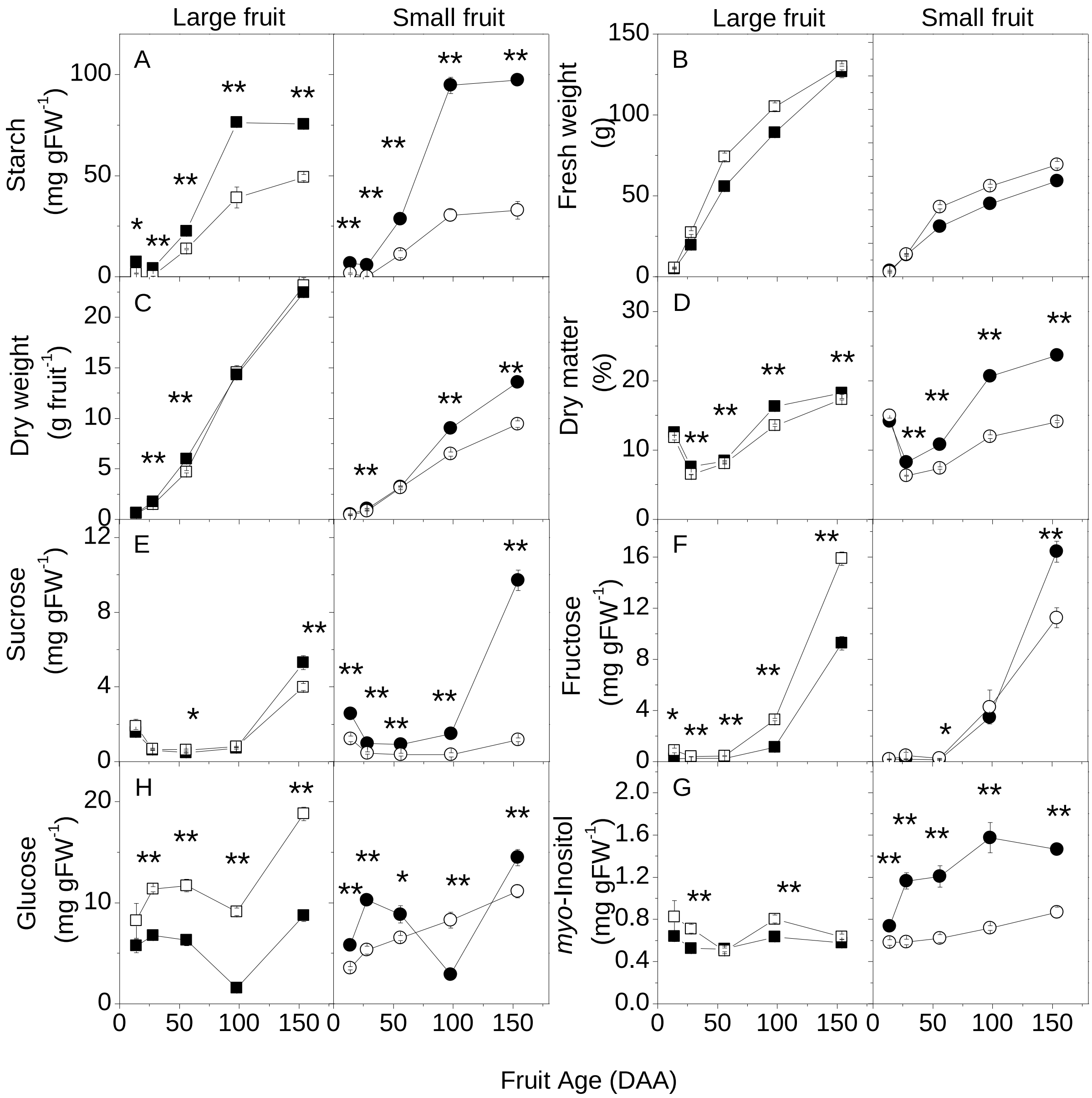

Supplementary Fig. S1. Changes in starch (A), physical measurements (B, fresh weight; C, dry weight; D, dry matter), and Ion Chromatography quantified soluble sugars (E, Suc; F, Fru; G, Glc) and the sugar-alcohol *myo*-inositol (H) in the outer pericarp during fruit development *of Actinidia deliciosa* genotypes (2009 samples). Four genotypes were examined: Genotype 3, large fruit and high starch (G3, close square); Genotype 25, large fruit and low-starch (G25, open square); Genotype 30, small fruit and high starch (G30, close circle), Genotype 17, small fruit and low-starch (G17, open circle). For each developmental stage, *t*-test significance levels are reported: *, p<0.05; **,

$p<0.01$; ***, $p<0.001$; ****, $p<0.0001$. Values are means of six biological replicates ±SEM.

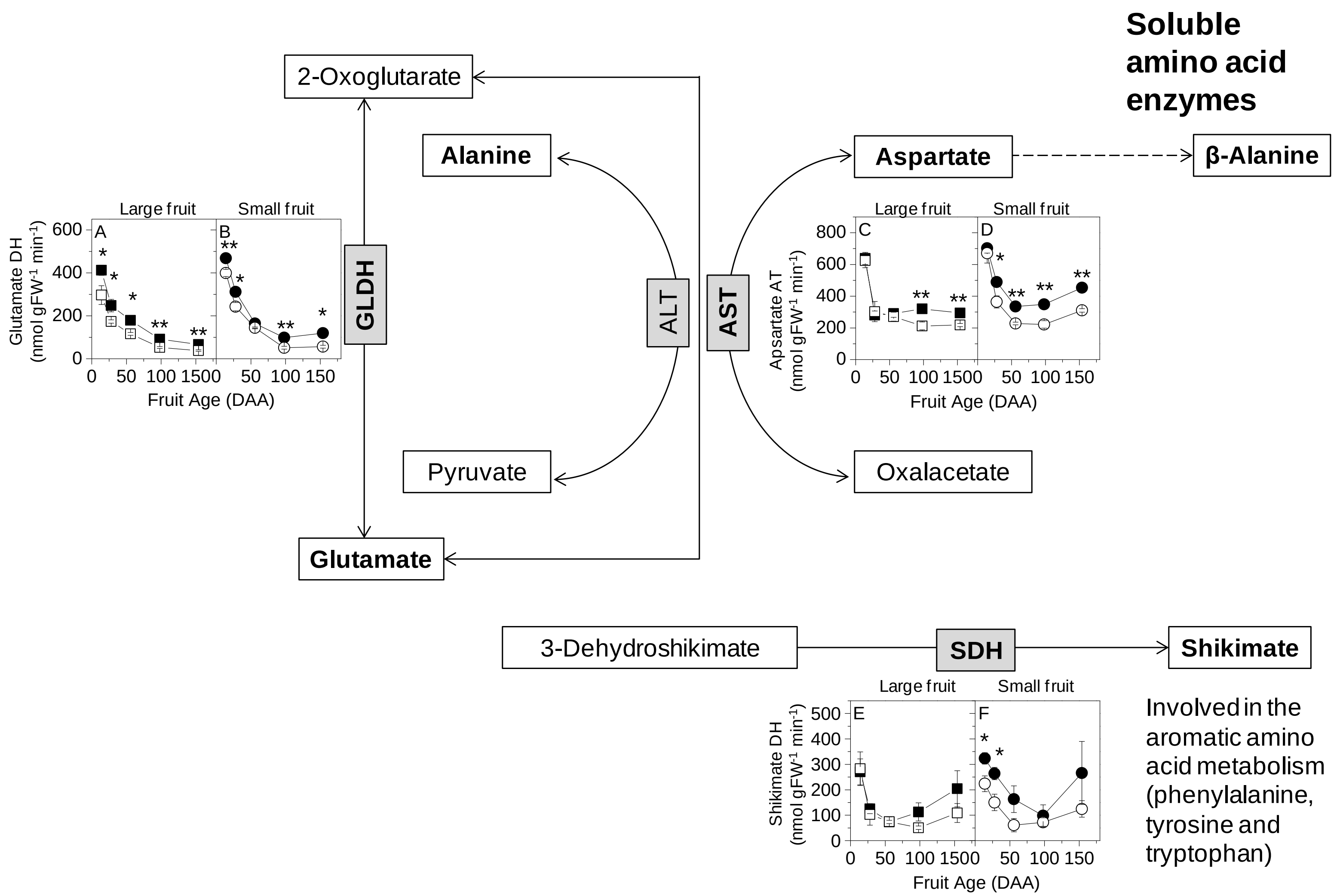

Supplementary Fig. S2. Kiwifruit soluble enzyme activities associated with amino acid metabolism. The graph for each enzyme activity is placed in proximity to the enzyme in the simplified pathway chart: A-B, Glutamate dehydrogenase; C-D,

Aspartate aminotransferase; E-F, Shikimate dehydrogenase. *t*-test significance levels are reported: *, *p*<0.05; **, *p*<0.01. Where the symbol is missing, the difference is not statistically significant. Genotype codes: G3, (high-starch, large fruit; close square); G17, (low-starch, small fruit; open circle); G25, (low-starch, large fruit; open square); G30, (high-starch, small fruit; close circle). Enzyme codes: ALT, alanine aminotransferase; AST, aspartate aminotransferase; GLDH, glutamate dehydrogenase; SDH, shikimate dehydrogenase. Fruit age is in days after anthesis (DAA).Values are means of 4 biological replicates ± SE of the mean. Data refer to 2009 outer pericarp samples. White boxes: metabolites; grey boxes: enzymes; bold text: measured metabolites.

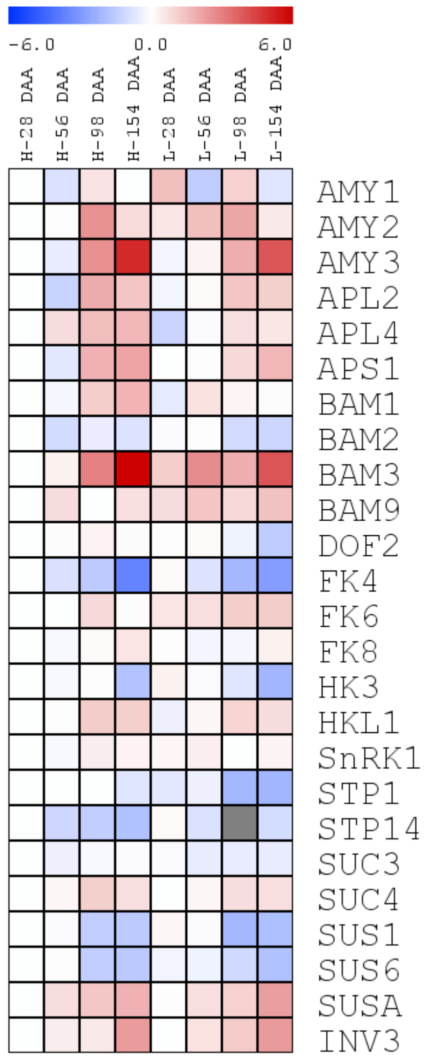

Supplementary Fig. S3. Heat map of fruit developmental transcript variations in ten *Actinidia deliciosa* genotypes for a selection of carbohydrate metabolism genes. Genotypes were classified according to the fruit starch concentration (H, high-starch; L, low-starch). Genes codes: α-amylases (*AMY1*, *AMY2* and *AMY3*), ADP-glucose pyrophosphorylases (*APL2*, *APL4* and *APS1*), β-amylase (*BAM1*, *BAM2*, *BAM3* and *BAM9*), DOF zinc finger (*DOF2*), fructokinase (*FK4*, *FK6* and *FK8*), hexokinase (*HK3* and *HKL1*), sucrose non-fermenting kinase (*SnRK1*), monosaccharide transporter (*STP1* and *STP14*), sucrose transporter (*SUC3* and *SUC4*), sucrose synthase (*SUS1*, *SUS6* and *SUSA*), and vacuolar invertase (*INV3*). Values are averages of five high-starch and five low-starch genotypes. Samples used in this study were whole fruit tissues collected in 2007. Housekeeping gene: protein phosphatase 2A (*PP2A*).

Data were normalised to the high-starch genotypes at 28 days after anthesis (DAA).

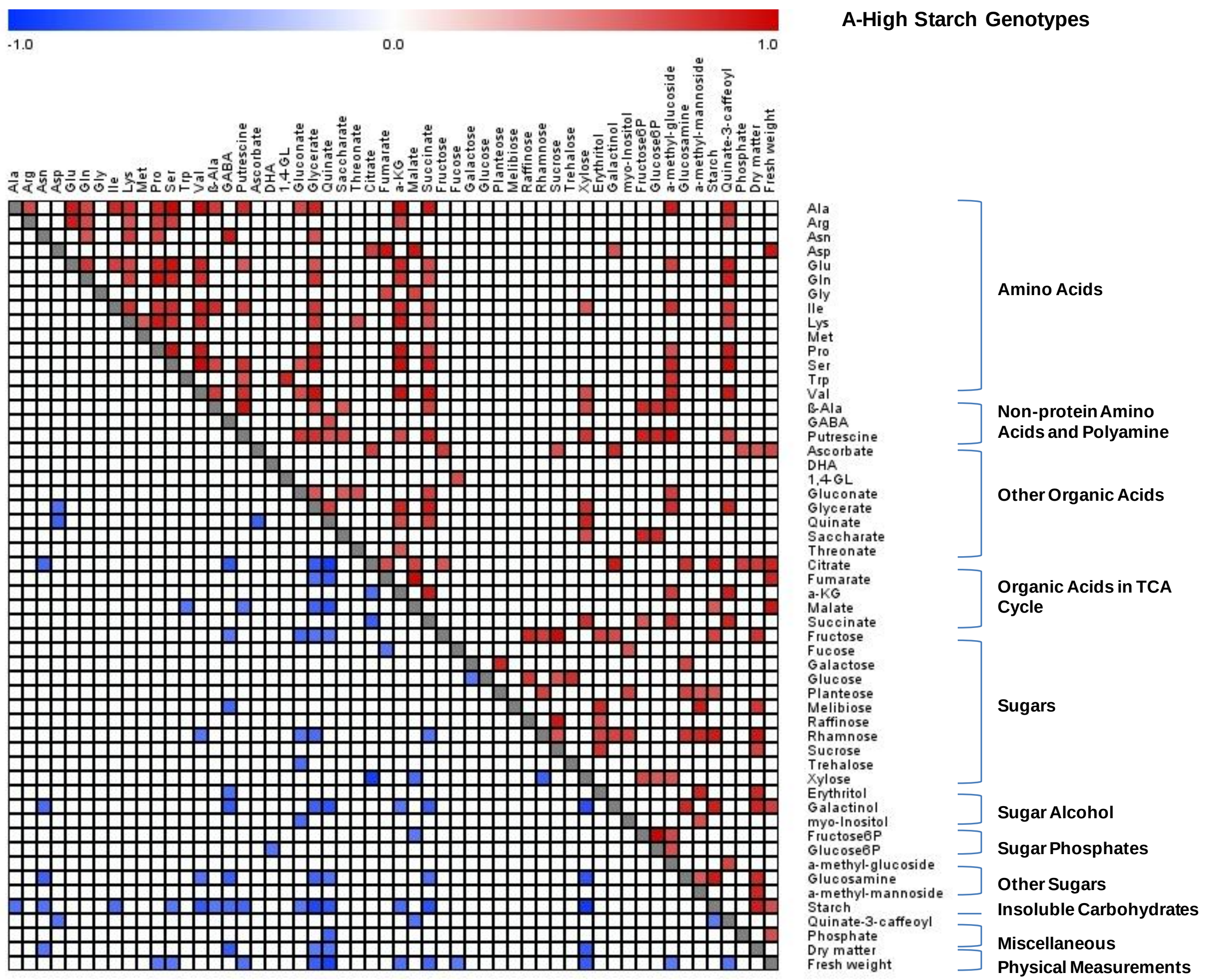

Supplementary Fig. S4. Metabolite correlation matrix during fruit development of kiwifruit berries. A, high-starch genotypes (G3 and G30); B, low-starch genotypes (G25 and G17). The significance threshold of *p*<0.05 was used (white

squares were not statistically significant). The 51 metabolites identified by GC-TOF-MS were subdivided into the following classes: amino acids (Ala, Trp, Arg, Gly, Gln, Ile, Pro, Asp, Ser, Asn, Val, Met, Lys, Glu); non-protein amino acids and polyamine (putrescine, β-Ala, GABA); other organic acids (threonate, quinate, DHA, gluconate, ascorbate, glycerate, saccharate, 1,4-GL); organic acids in the TCA cycle (succinate, malate, citrate, α-KG, fumarate); sugars (Glc, Fru, Suc, Gal, planteose, melibiose, Rha, Tre, Xyl, Fuc, raffinose); sugar-alcohols (*myo*-inositol, erythiritol, galactinol); phosphate sugars (Glc6P, Fru6P); miscellaneous (phosphate, quinate 3-caffeoyl); other sugars (α-methyl glucoside, α-methyl mannoside, glucosamine). Starch (insoluble carbohydrates) and physical measurements (fresh weight and dry matter) were also analysed. DHA, dehydroascorbate; GABA, γ-aminobutyric acid; 1,4-GL, glucaric acid 1,4-lactone; α-KG, α-ketoglutarate. Data refer to 2009 outer pericarp samples.

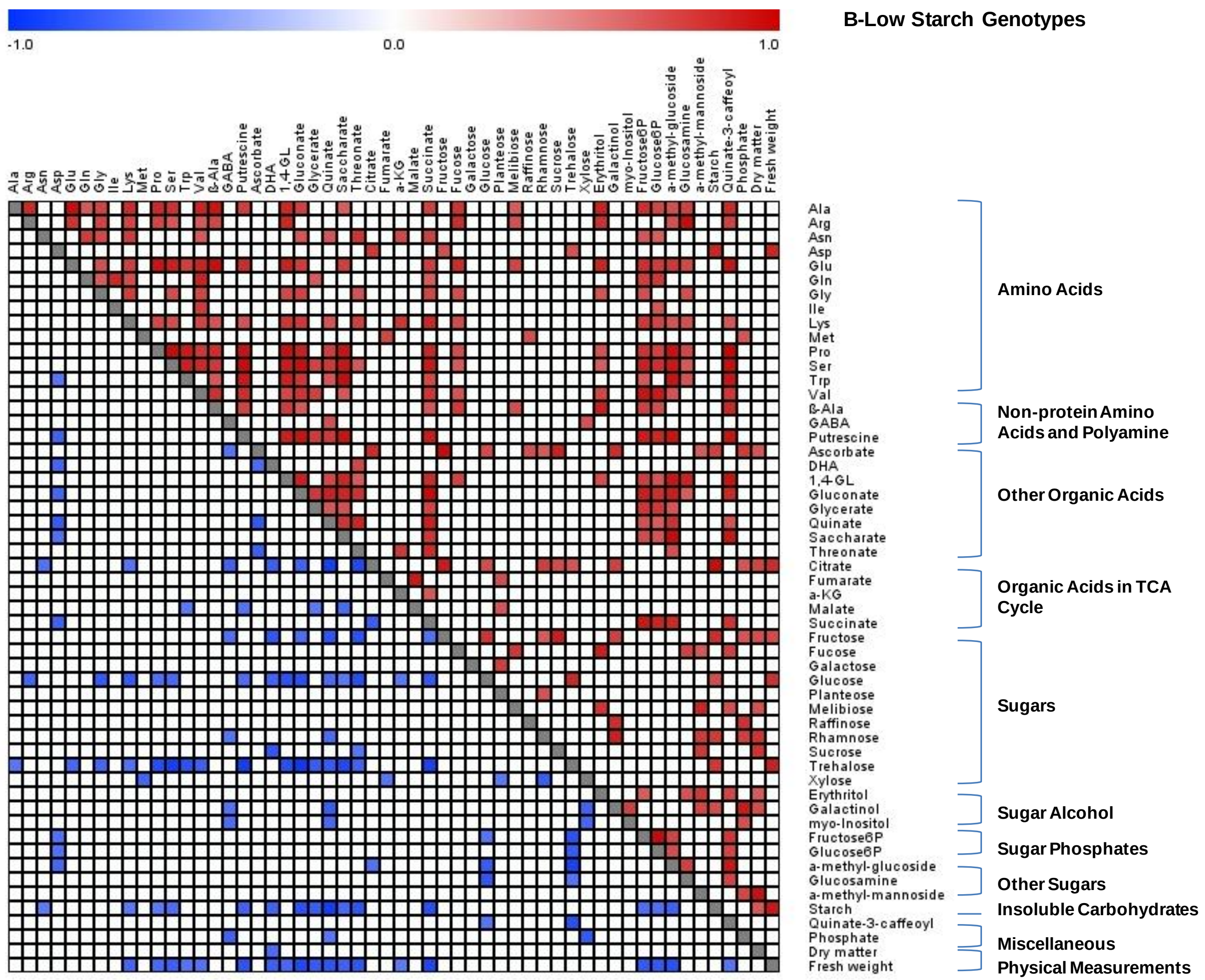

Supplementary Fig. S4. (Continued.).

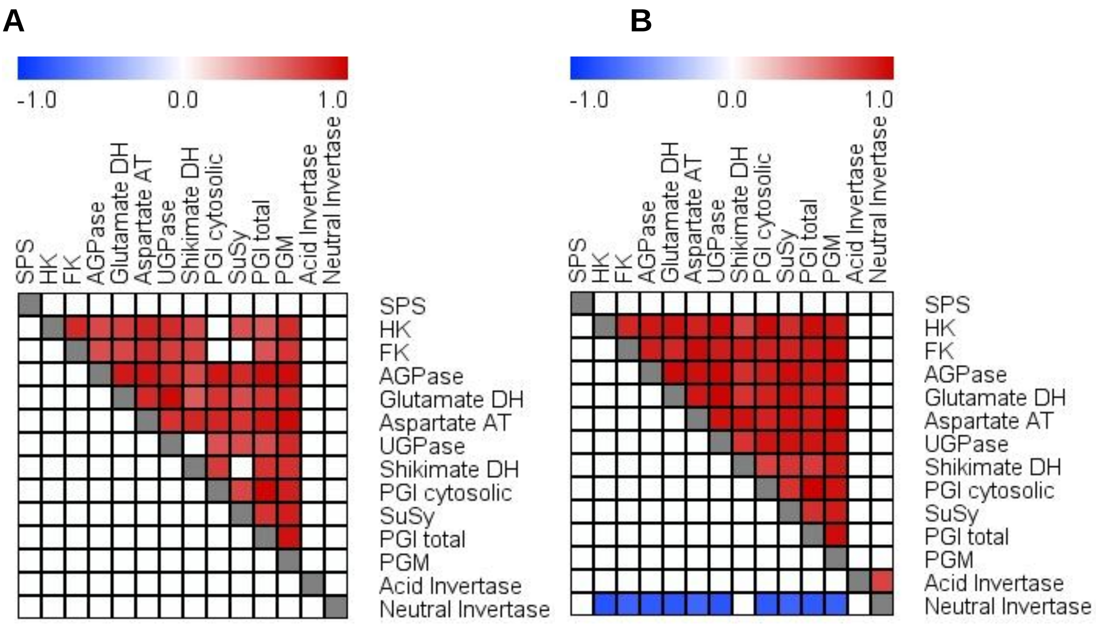

Supplementary Fig. S5. Enzyme activity correlation matrix during fruit development of kiwifruit berries. A, high-starch genotypes (G3 and G30); B, low-starch genotypes (G25 and G17). The significance threshold of *p*<0.05 was used (white squares were not statistically significant). Enzyme codes: AGPase, ADP-glucose pyrophosphorylase; Aspartate AT, aspartate aminotransferase; FK, fructokinase; HK, hexokinase; Glutamate DH, glutamate dehydrogenase; PGM, phosphoglucomutase; PGI, phoshoglucose isomerase; shikimate DH, shikimate dehydrogenase; SPS, sucrose phosphate synthase; SuSy, sucrose synthase; UGPase, UDP-glucose pyrophosphorylase. Data refer to 2009 outer pericarp samples.

A

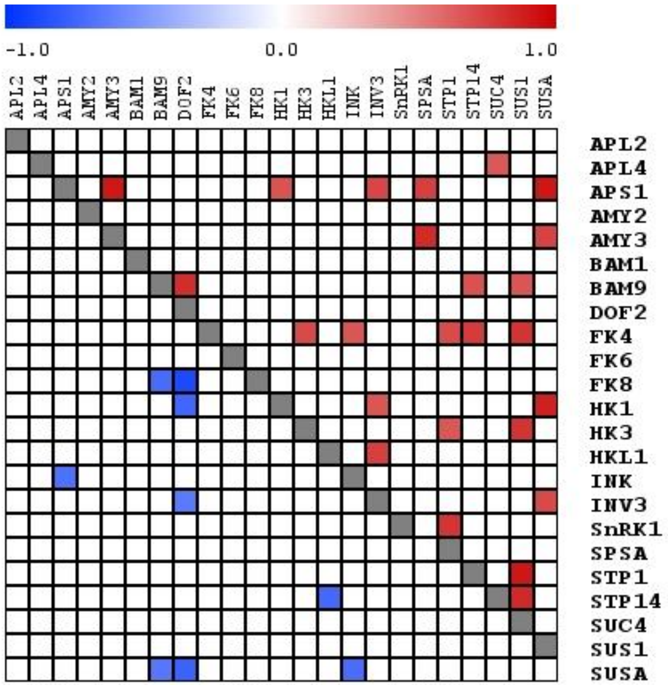

B

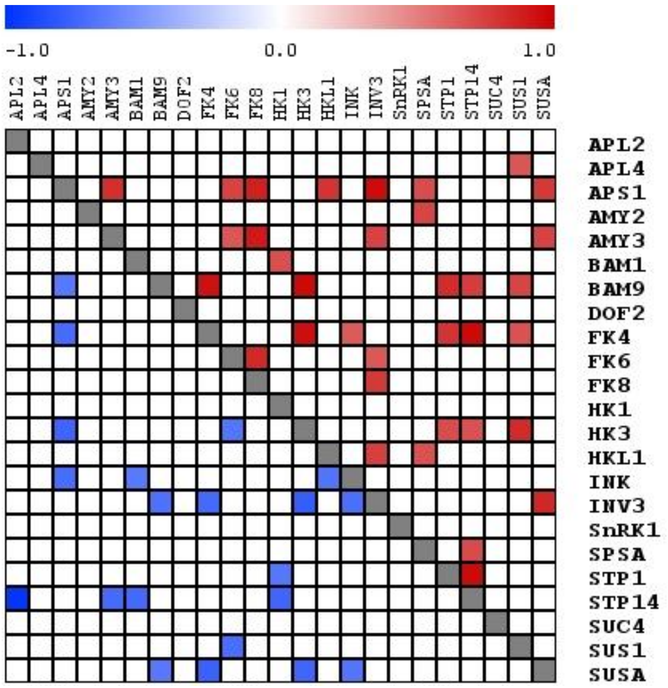

Supplementary Fig. S6. Transcript correlations in fruit outer pericarp of *Actinidia deliciosa* genotypes (2009 samples): high-starch genotypes (A, genotype 3 and genotype 30) and low-starch genotypes (B, genotype 25 and genotype 17). Only significant correlations are shown (*p*<0.05). Gene codes: ADP-glucose pyrophosphorylases (*APL2*, *APL4* and *APS1*), α-amylases (*AMY2* and *AMY3*), β-amylase (*BAM1* and *BAM9*), DOF zinc finger (*DOF2*),

fructokinase (*FK4*, *FK6* and *FK8*), hexokinase (*HK3* and *HKL1*), neutral invertase (*INK*), vacuolar invertase (*INV3*), sucrose non-fermenting kinase (*SnRK1*), sucrose phosphate synthase (*SPSA*), monosaccharide transporter (*STP1* and *STP14*), sucrose transporter (*SUC4*) and sucrose synthase (*SUS1* and *SUSA*).

A

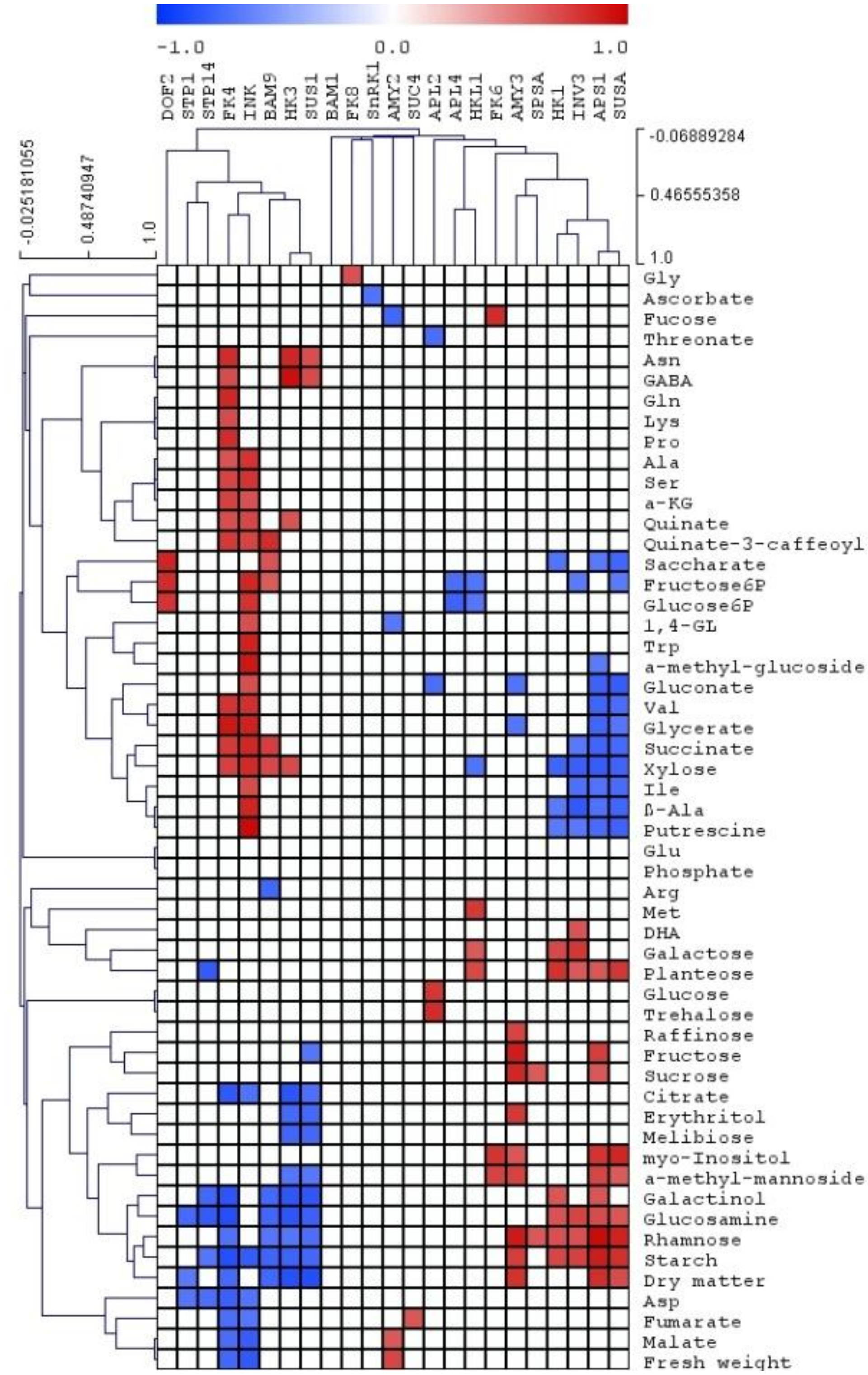

Supplementary Fig. S7. Hierarchical cluster analysis of metabolite to transcript correlations in fruit outer pericarp of *Actinidia deliciosa* genotypes (2009 samples): high-starch genotypes (A, genotype 3 and genotype 30) low-starch genotypes (B, genotype 25 and genotype 17). Only significant correlations are shown (*p*<0.05). Gene codes: ADP-glucose pyrophosphorylases (*APL2*, *APL4*

and *APS1*), α-amylases (*AMY2* and *AMY3*), β-amylase (*BAM1* and *BAM9*), DOF zinc finger (*DOF2*), fructokinase (*FK4*, *FK6* and *FK8*), hexokinase (*HK3* and *HKL1*), neutral invertase (*INK*), vacuolar invertase (*INV3*), sucrose non-fermenting kinase (*SnRK1*), sucrose phosphate synthase (*SPSA*), monosaccharide transporter (*STP1* and *STP14*), sucrose transporter (*SUC4*) and sucrose synthase (*SUS1* and *SUSA*). DHA, dehydroascorbate; GABA, γ-aminobutyric acid; 1,4-GL, glucaric acid 1,4-lactone; α-KG, α-ketoglutarate.

B

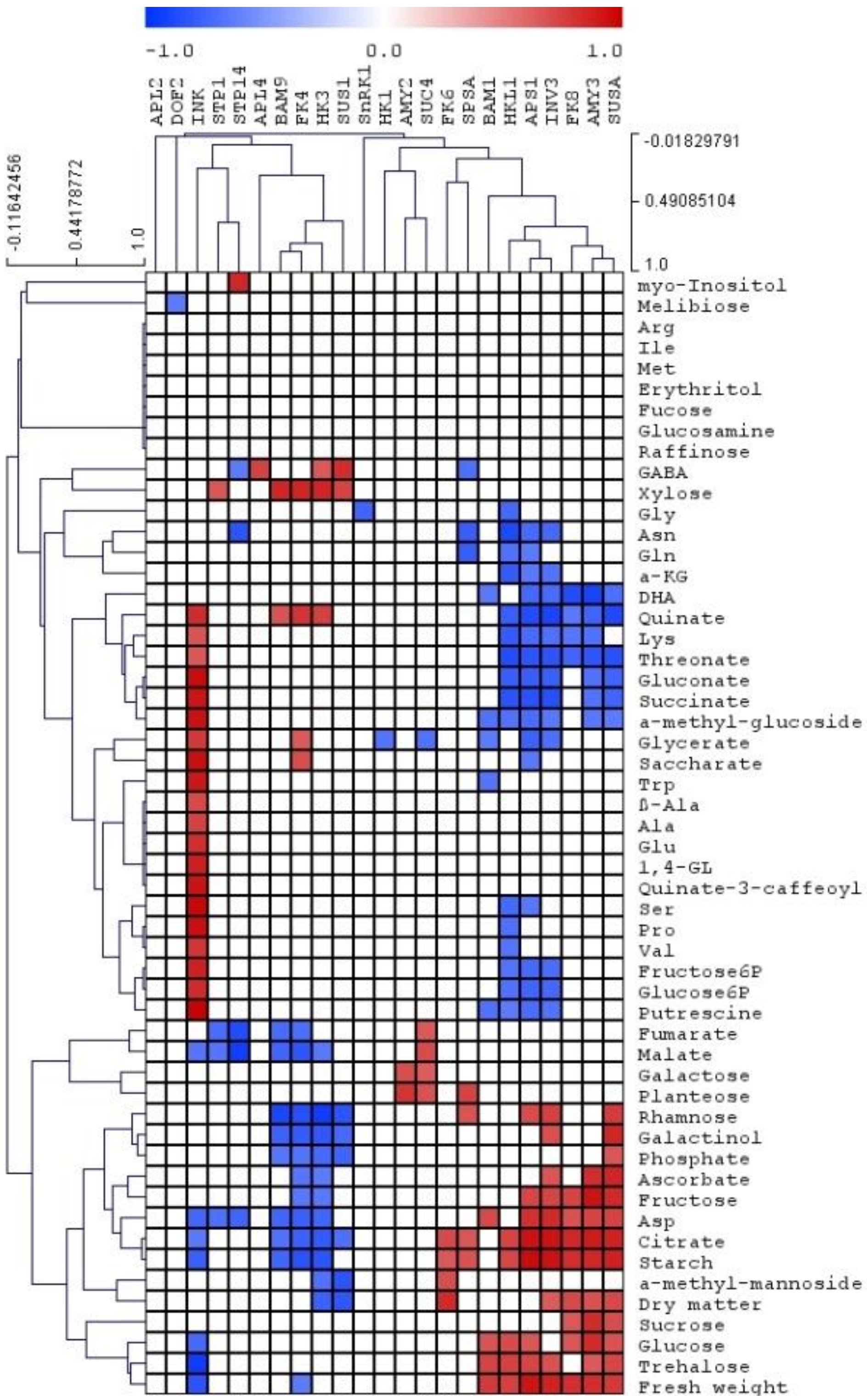

Supplementary Figure S7. (Continued.).

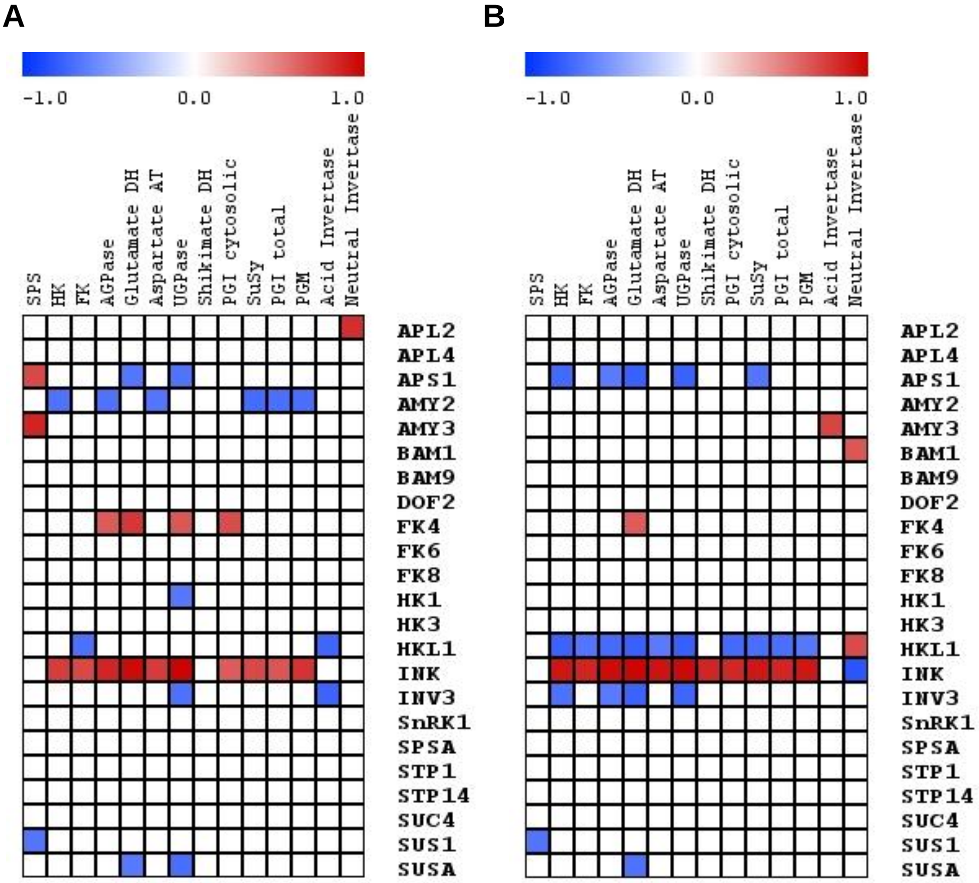

Supplementary Fig. S8. Transcript to enzyme correlations in fruit outer pericarp of *Actinidia deliciosa* genotypes (2009 samples): high-starch genotypes (A, genotype 3 and genotype 30) low-starch genotypes (B, genotype 25 and genotype 17). Only significant correlations are shown (*p*<0.05). Gene codes: ADP-glucose pyrophosphorylases (*APL2*, *APL4* and *APS1*), α-amylases (*AMY2* and *AMY3*), β-amylase (*BAM1* and *BAM9*), DOF zinc finger (*DOF2*), fructokinase (*FK4*, *FK6* and *FK8*), hexokinase (*HK3* and *HKL1*), neutral invertase (*INK*), vacuolar invertase (*INV3*), sucrose non-fermenting kinase (*SnRK1*), sucrose phosphate synthase (*SPSA*), monosaccharide transporter (*STP1* and *STP14*), sucrose transporter (*SUC4*) and sucrose synthase (*SUS1* and *SUSA*). Enzyme codes: AGPase, ADP-glucose pyrophosphorylase; Aspartate AT, aspartate aminotransferase; FruK, fructokinase; HK, hexokinase; Glutamate DH, glutamate dehydrogenase; PGM, phosphoglucomutase; PGI, phoshoglucose isomerase; shikimate DH, shikimate dehydrogenase; SPS, sucrose phosphate synthase; SuSy, sucrose synthase; UGPase, UDPglucose pyrophosphorylase.

References

Fung RWM, Langenkämper G, Gardner RC, MacRae E. 2003. Differential expression within an SPS gene family. *Plant Science* 164, 459-470.

Gibon Y, Blaesing OE, Hannemann J et al. 2004. A robot-based platform to measure multiple enzyme activities in *Arabidopsis* using a set of cycling assays: comparison of changes of enzyme activities and transcript levels during diurnal cycles and in prolonged darkness. *Plant Cell* 16, 3304-3325.

Nardozza S, Boldingh HL, Richardson AC, Costa G, Marsh H, MacRae EA, Clearwater MJ. 2010. Variation in carbon content and size in developing fruit of *Actinidia deliciosa* genotypes. *Functional Plant Biology* 37, 545–554.

Richardson A, Boldingh H, McAtee P, Gunaseelan K, Luo Z, Atkinson R, David K, Burdon J, Schaffer R. 2011. Fruit development of the diploid kiwifruit, *Actinidia chinensis* 'Hort16A'. *BMC Plant Biology* 11, 182.

Steinhauser M-C, Steinhauser D, Koehl K, Carrari F, Gibon Y, Fernie AR, Stitt M. 2010. Enzyme activity profiles during fruit development in tomato cultivars and *Solanum pennellii*. *Plant Physiology* 153, 80-98.
